# Vale do Côa rock art as linked open data: Application profile, schemas, and data

**DOI:** 10.12688/openreseurope.21901.1

**Published:** 2026-03-03

**Authors:** Ana Baptista, Inês Miguel, Juliana Galvão, Natália Botica

**Affiliations:** 1 Information Systems Department / ALGORITMI Research Center, University of Minho, Guimarães, 4804 - 533, Portugal; 2 Archaeology Unit, University of Minho, Braga, 4710-229, Portugal

**Keywords:** Archeology, Rock Art, Côa Valley, Linked Open Data (LOD), Resource Description Framework (RDF), Application Profile

## Abstract

**Background:**

The Côa Valley Archaeological Park, a UNESCO World Heritage site and the world's most extensive example of open-air Paleolithic art, holds crucial data on its over 1,200 engraved rocks in the University of Minho's proprietary 2ArchIS database. This article addresses the need to transition this restricted information into an openly accessible format to benefit the broader scientific community.

**Methods:**

The work involved transforming the 2ArchIS data into Linked Open Data (LOD). This process began with analyzing the existing data model and content. Subsequently, a series of standards-based artifacts were developed: a Dublin Core Tabular Application Profile (DCTAP), an RDF Schema (RDFS) vocabulary, a Simple Knowledge Organization System (SKOS) controlled vocabulary, and a Shape Expressions (ShEx) schema for data validation. The data exported from 2ArchIS was transformed into RDF using OpenRefine, validated against the ShEx schema, and finally hosted on a triplestore.

**Results:**

The project successfully generated a complete LOD dataset of the Vale do Côa engravings that is openly available. All resulting developmental artifacts, the DCTAP, RDFS and SKOS vocabularies, and the ShEx schema, are openly available for the scientific community's use and reuse.

**Conclusions:**

This initiative has significantly broadened access to vital Paleolithic art data by publishing it as Linked Open Data, thereby enhancing its research potential. Future efforts will include creating a user-friendly interface to help non-technical users query the triplestore.

## Introduction

Vale do Côa (Côa Valley, in English) is an Archaeological Park in the Guarda district, Portugal. The park has a considerable area with more than 1200 engraved rocks. It is considered the world's most extensive set of Paleolithic art in the open and has been part of the UNESCO World Heritage list since 1998. Most of those 1200 engraved rocks belong to the Neolithic and Iron Age periods. ^1, 2^

Global initiatives promoting open, structured, and reusable data have significantly influenced the digital transformation of Cultural Heritage (CH). The digital transformation of cultural collections has strengthened preservation practices and opened new opportunities for research, education, and public engagement. ^3, 4^ This paradigm shift has been accompanied by international initiatives that promote open, structured data, ensuring that heritage knowledge remains accessible and reusable sustainably. Movements such as Open GLAM and Collections as Data advocate for the responsible sharing and ethical reuse of cultural data. This approach is exemplified by the principles articulated in documents such as the Vancouver Statement on Collections as Data. ^5^ These initiatives align closely with the FAIR principles (Findable, Accessible, Interoperable, and Reusable), ^6^ which enhance machine-actionable data discovery and usage while supporting human reuse, and with the CARE principles (Collective Benefit, Authority to Control, Responsibility, Ethics), which provide a foundational approach to Indigenous Data Governance. ^7^

This research was developed within the scope of the RARAA project, funded by the Portuguese Foundation for Science and Technology (FCT). The project’s team explored the Côa Valley in search of Iron Age rock art, aiming to increase knowledge of the art and the communities that created it. The team did fieldwork searching specific areas, such as Vermelhosa, Vale de José Esteves, and Meijapão. ^8^

All the data gathered by the RARAA project is stored in a relational database (RDB) called 2ArchIS. Although the RDB is beneficial and widely used by the RARAA project and the Archaeology Unit of the University of Minho (UAUM) team, the researchers would like to be able to relate their data to those of other research teams and, conversely, to be able to share their data with other researchers or interested citizens for use and reuse. In other words, the RARAA team wanted its data to be open and semantically interoperable, that is, to be Linked Open Data (LOD).

Linked Data refers to rules and good practices for publishing structured data on the Web (Heath & Bizer, 2011). As Tim Berners-Lee (as cited by ^9^) puts it, “Linked Data is the Semantic Web done right, and the Web done right”. LOD is Linked Data that is open for use and reuse. “Open means anyone can freely access, use, modify, and share for any purpose (subject, at most, to requirements that preserve provenance and openness)”. ^10^ One of the most relevant technologies for Linked Data is the Resource Description Framework (RDF), which is used to represent information on the Web. ^11^

The goal of the project was, then, to transform data in an RDB into LOD and make it available for use and reuse, adhering to principles that promote structured, reusable, and interconnected data. That is expected to allow the integration of RARAA’s data with data from other research teams and enable broader use and reuse by the academic community and the general public. To this end, we first created an application profile and related artifacts, then used them to expose the 2ArchIS data as Linked Data. An application profile “defines metadata usage for a specific application”. ^12^

This article presents the entire process of converting data from an RDB to LOD, from the design of the application profile to its implementation, development of Shape Expressions (ShEx) schema, development of new properties and classes, structuring and encoding of controlled vocabularies, transformation of the data, and making it available in the triplestore. ShEx “is a language for describing RDF graph structures”. ^13^ An RDF graph may be tested against a ShEx schema to yield a validation result. The ShEx schema prescribes the conditions that RDF graphs must meet to be considered conformant. Triplestores are “tools that can be installed to serve as RDF databases”. ^14^

Despite many advancements in LOD solutions for CH, a significant gap persists in the domain of rock art data, where no comprehensive LOD solution exists to address the unique challenges of describing and linking data on motifs, scenes, and their archaeological context. In many cases, LOD is created from scratch, but there are also numerous cases where the objective is to open and link data that already exists in closed environments. This work, therefore, describes a complete methodological pipeline to open existing data in a closed environment and proposes a set of artifacts specifically designed for rock art documentation.

As a contribution to other researchers, we make explicit the process we followed to generate LOD from closed data as well as the main decisions taken across the process. This process follows and aligns with current best practices for publishing data on the Web, including the FAIR principles and the Data on the Web Best Practices (DWBP). ^6, 15, 16^ Furthermore, we reflect on the overall process and on the use of the Dublin Core Tabular Application Profile (DCTAP), a recent construct designed to support the development and representation of application profiles. ^12^ As a contribution to professionals, we present several artifacts and the resulting Linked Data openly available for use and reuse.

We expect this work to be helpful to researchers and professionals in the fields of Linked Data and metadata, particularly in rock art or archaeology, as well as to students seeking guidance on an effective workflow for converting relational data to Linked Data.

Beyond this introduction, the article is organized into the following sections: Related Work (section 2); Information about the 2ArchIs database (section 3); Methodological Procedures (section 4); Results (section 5); Conclusions and Future Work (section 6).

## Related work

In this work, we analyzed the schemas and vocabularies produced across various projects and initiatives, some specific to CH and others cross-domain. Furthermore, we analyzed development processes in projects of this nature that aim to open a CH collection as LOD. The following subsections reflect the results of these analyses.

### Ontologies and vocabularies for cultural heritage and archaeology

To contextualize our approach, we first identify several key initiatives that have served as guiding references in the vast landscape of Cultural Heritage Linked Open Data (CH LOD). Among these are the Advanced Research Infrastructure for Archaeological Dataset Networking in Europe (ARIADNE) and its successor, ARIADNEplus; the International Committee for Documentation of the International Council of Museums Conceptual Reference Model (CIDOC CRM); Archaeological Finds on the Semantic Web (FindSampo); and the Europeana Data Model (EDM).

In our case, the most relevant are ARIADNE and ARIADNEplus, which focus on developing semantically interoperable archaeological data repositories to make data more accessible and easily searchable for researchers. ^17^ By enabling partners to integrate their datasets within a common infrastructure, ARIADNE successfully connected archaeological data from multiple sources, ^18^ making them available as LOD for use and reuse. ARIADNE ran between 2012 and 2016. ^19^

The backbone of the ARIADNE Reference Model is CIDOC CRM, described as “a global extensible schema in the form of a formal ontology that allows for integration without loss of meaning”. ^20^ The ARIADNE Catalogue Data Model (ACDM) was developed to describe the various standards, formats, and services already used by content providers. This model was one of the pillars for developing the AO-CAT ontology within the ARIADNEplus project. ^21^ This ontology comprises 66 properties and 22 classes with minimal constraints to avoid making their application too restrictive. ARIADNEplus was an extension of ARIADNE that ran from 2018 to 2022. Both projects use CIDOC CRM as their reference model. ^17, 20^ The ARIADNE Research Infrastructure AISBL (ARIADNE RI) was established by the end of 2022, and a LOD triplestore was established, providing access to 3.5 million resources encompassing the archaeology and CH of over 40 countries across four continents. ^19, 22^

CIDOC CRM is widely recognized as both a theoretical and practical framework for integrating information in the field of CH. ^23^ It provides a “shared understanding” of CH information, acting as a “semantic glue” to mediate CH data originating from a variety of sources. ^24, 25^ It is a “living standard” consisting of a base ontology (CRMbase) complemented by several modular extensions, two of which are for archaeological data: CRMba for archaeological buildings and CRMarchaeo for excavations. The latest stable release of CIDOC CRM (version 7.1.3) defines 81 classes and 160 properties, while CRMArchaeo ontology consists of 10 classes and 32 properties. ^25, 26^

Another relevant ontology is MAO/TAO [1], developed within the FindSampo initiative ( *Archaeological Finds on the Semantic Web*), a *portal and linked open data service* that provides access to information about Finnish archaeological finds, particularly those discovered and reported by the public. ^27^ The MAO/TAO ontology, maintained by the Finnish Heritage Agency, is particularly well-suited for indexing and describing materials related to museums and art. ^28^ It results from the integration of two domain ontologies: MAO, focused on museum collections, and TAO, related to applied arts. ^29^ This ontology was valuable to our project, especially during the early stages of controlled vocabulary design, as it provided concrete examples of terminology alignment, hierarchical structure, and naming conventions in the museum and archaeological domains.

Finally, the Europeana Data Model aims to be “an integration medium for collecting, connecting and enriching the descriptions provided by Europeana's content providers”. ^30^ Europeana provides access to Europe's digital CH, ^31^ including libraries, museums, archives, and galleries. It uses 18 classes and 77 properties from several namespaces, mainly EDM, DC Terms, and Object Reuse and Exchange (ORE). The EDM is key in the Common European Data Spaces [2], particularly for CH [3]. ^32^ Common European Data Spaces are a European Union initiative that aims to make more data available for use and reuse through a “trustworthy and secure environment for the benefit of European businesses and citizens”. ^33^

### General-purpose metadata Ontologies and properties' vocabularies

Using general-purpose vocabulary schemes can enhance semantic interoperability across domain and application boundaries, contributing to a more direct relationship between datasets from different knowledge domains. In our case, we have, whenever possible, tried to adopt terms from vocabularies specific to archaeology or CH, such as those identified in the previous section. However, when this was not feasible, we relied on general-purpose vocabularies, either using their terms directly or employing them as properties or classes to relate to our own. Some schemas, due to their broad scope and relevance to our project, must be mentioned explicitly.

The Dublin Core Metadata Initiative (DCMI) Terms (DC Terms) specification is a DCMI recommendation. Among its properties are the well-known 15 elements from the Dublin Core Metadata Element Set, often just called Dublin Core. DC Terms has many more properties; some are even sub-properties of other DC Terms properties (e.g., issued is a sub-property of date). These properties are used in various domains and applications, including digital repository platforms (e.g., DSpace [4]) and the Data Catalogue Vocabulary [5] (DCAT), whose application profile (DCAT-AP [6]) is key for describing datasets in the Common European Data Spaces. ^34^ Several DC Terms properties and classes have been published as the ISO standard 15836-2:2019. ^35^

DCAT is “an RDF vocabulary designed to facilitate interoperability between data catalogs published on the Web”. ^36^ It enables the description of datasets and data services, improving their discovery and access. A good description of datasets also enhances their interoperability and, therefore, their use and reuse, as advocated by the FAIR principles. DCAT-AP, an application profile of DCAT, is used to describe data from European public organizations. It is used in the European Data Portal [7] and the Common European Data Spaces.

DBpedia [8] is a “crowd-sourced community effort to extract structured content from the information created in various Wikimedia projects”. ^37, 38^ It provides structured data “for more than 100 Wikipedia language editions as well as Wikimedia Commons, has a mature ontology and a stable and thorough Linked Data publishing lifecycle”. ^39^ At its core is the DBpedia ontology (DBO). ^40^ DBO is a crowd-sourcing effort resulting in a cross-domain ontology that currently contains 768 classes and 3,000 properties, ^40^ many of which are used in other projects for interoperability and reusability. Another DBpedia namespace, with the prefix dbp, represents “properties extracted from the raw infobox extraction,” whose data is considered lower quality than the DBO's. ^41^ There are other namespaces in DBpedia that have various purposes. Examples are dbr [9], for entities or resources, and dbt, for properties from infobox templates [10].

Like DBpedia, Wikidata [11] is a collaborative project associated with the Wikimedia Foundation [12]. Wikidata and DBpedia both overlap and complement each other. All DBpedia data is extracted from Wikimedia projects, ^39^ while Wikidata's data is created collaboratively and feeds projects such as Wikipedia and others. ^42^ Its open knowledge base acts as a central storage of structured data for Wikimedia projects. ^43^ The data is available in several formats under a Creative Commons Universal License [13].

Schema.org [14] is “a collaborative, community activity with a mission to create, maintain, and promote schemas for structured data on the Internet, on web pages, in email messages, and beyond”. ^44^ It is an initiative founded by Google, Microsoft, Yahoo, and Yandex that provides a schema whose terms are used extensively on the Web. It currently consists of 817 types and 1518 properties, among other kinds of terms. ^45^ Because it may be of interest to data providers and catalog publishers, version 3 of the DCAT specification proposes an alignment with Schema.org. ^36^

There are other schemas that, although not general-purpose, are applicable across various domains, such as those for geographic data or units of measure. Examples of this type of schema include GeoNames [15] or the Getty Thesaurus of Geographic Names Online [16]. GeoNames is a service based on a database containing over 25 million geographic names available for download free of charge under a Creative Commons Attribution license [17]. ^46^ Each geographic name in GeoNames has a unique identifier ^46^ that can be used in other Linked Data projects. The names are also related to each other. The same is true of the TGN. The TGN includes more than 5 million geographic names available in various formats, including several LOD formats. The Open Data Commons Attribution License (ODC-By) 1.0 [18] is the license used. On its website, TGN describes other geographic initiatives for Linked Data, including GeoNames, and compares them in some respects, including geographical coverage, temporal coverage (including historical data), and data quality. ^47^ Many other namespace schemas exist, which may be found at services such as the Linked Open Vocabularies (LOV) [19].

### Linked data development processes in cultural heritage

Over the years, several projects have focused on generating Linked Data from CH data stored in RDBs, spreadsheets, or even from unstructured data. Some of these projects make extensive use of ontologies, with new developments addressing gaps in existing models. In contrast, others focus more on property and value vocabularies, some reusing them, others establishing mappings to relate new terms to existing ones. Many projects also draw inspiration from methods, models, tools, and techniques developed in other disciplines. For example, De Boer and colleagues ^48^ present a process “for ingesting, converting, and linking cultural heritage metadata into Linked Data” that, according to the authors, was designed to maintain the richness of the original metadata, as opposed to practices of mapping to specific schemas where they claim part of this richness to be lost. Several tools support this process, which had the following steps: 1) XML ingestion; 2) Direct transformation to ‘crude’ RDF; 3) RDF restructuring; 4) Creation of a metadata mapping schema; 5) Alignment of vocabularies with external sources; and 6) Publishing as Linked Data. Hong and colleagues ^49^ developed a transformation framework to migrate large volumes of data from RDBs to ontology-based datasets. They created a scripting language “which allows the specification of complex transformation rules from data objects to ontologies”. This language was put into practice using an extended version of CIDOC-CRM. Daquino and colleagues ^50^ describe the work carried out to make the photo archive Zeri available as Linked Data through new ontology extensions to existing models. They then mapped the archive's descriptive elements into RDF using the ontologies they created and CIDOC-CRM with examples of usage. These developments enabled them to generate an RDF dataset that includes descriptions of approximately 50,000 entries from the Zeri Archive. Nishanbaev and colleagues ^51^ developed a feature comparison of tools, including OpenRefine, for generating geospatial RDF data. They also propose a methodology for producing linked geospatial data that consists of five steps: 1) Data preparation; 2) Mapping the data into the ontology; 3) Interlinking RDF datasets; 4) Storing RDF data; and 5) Geospatial Semantic Web app & LOD cloud. Carriero and colleagues ^52^ used and extended eXtreme Design to develop and validate ArCo, the knowledge graph of Italian CH, represented as Linked Data. eXtreme Design is an ontology design methodology inspired by eXtreme Programming, an agile software development methodology. The authors extended eXtreme Design to deal with Cultural Heritage ontology projects. Binding and colleagues ^53^ report on the development of Linked Data departing from exported data, where they mapped existing fields to CIDOC CRM and converted the data to RDF using a “template-based data conversion method”. They also encoded and published specific controlled vocabularies as Linked Data.

Our development process is generally aligned with some of the presented processes, but in some parts, we have taken different approaches. Firstly, we chose to use a generic open data repository, in this case the University of Minho's data repository, to store motifs’ data and images, as well as project outputs, and to generate persistent identifiers, in this case Digital Object Identifiers (DOIs), for each item. We created a record for each motif in the data repository, thereby assigning a DOI to each. In this same registry, we include other information, such as images, as well as tabular data. This way, each motif has a DOI, and its associated information is preserved in a stable, long-term repository. We also stored all developed artifacts in the open data repository, generating a DOI for each, thereby supporting their long-term digital preservation. Secondly, we chose to use DCTAP, aligning the development of the application profile with the most recent developments in DCMI, a leading international initiative in metadata. Detailed information about the entire process is presented in Section 4.

To the best of our knowledge, our project is the first to dedicate itself exclusively and in depth to making LOD for rock art available, developing a range of artifacts and the data itself. Our project aims to make a first contribution in this direction. Drawing on multiple sources, building on them, and expecting to contribute to the Linked Data community in archaeology and CH, this work presents an application profile, a namespace schema, a controlled vocabulary, a ShEx schema, and actual rock art data. In addition, it presents in detail the process that led to making this data openly available from an RDB.

## The 2ArchIS relational database

The rock art of the Côa Valley is an in situ heritage whose study contributes to a better understanding of the societies that created it. Analyzing artistic representations on rocks provides insights into ancient communities' beliefs, religious practices, social structures, rituals, and the environments in which they lived. The animal representations in rock engravings can offer clues about the environment and ecosystem in which these communities lived, providing information about extinct or changing fauna, climatic patterns, and ancient landscapes. Additionally, hunting scenes depicted in the art may indicate survival and subsistence techniques and strategies. Therefore, the study of rock art representations is a cross-disciplinary endeavor where data sharing is essential for a better understanding of the cultural landscape in which the engravings are situated. Consequently, their archaeological record, often unique and irreplaceable, represents a valuable heritage whose preservation is crucial, allowing for new interpretations and studies. Rock engravings, particularly susceptible to natural factors such as erosion, wind, rain, freeze-thaw cycles, and vandalism, underscore the need to safeguard this cultural legacy to study and preserve its memories.

However, safeguarding archaeological data without FAIR data requirements is not sufficient. It is imperative to build trust in the data and facilitate its reuse in subsequent studies, integrating data and developing consistent results. Rock art documentation should be structured to ensure interoperability and reusability, following the FAIR principles. This endeavor involves standardizing records, establishing controlled vocabularies, and attaching metadata to ensure the data's quality and reliability. ^8^

**Figure 1.**
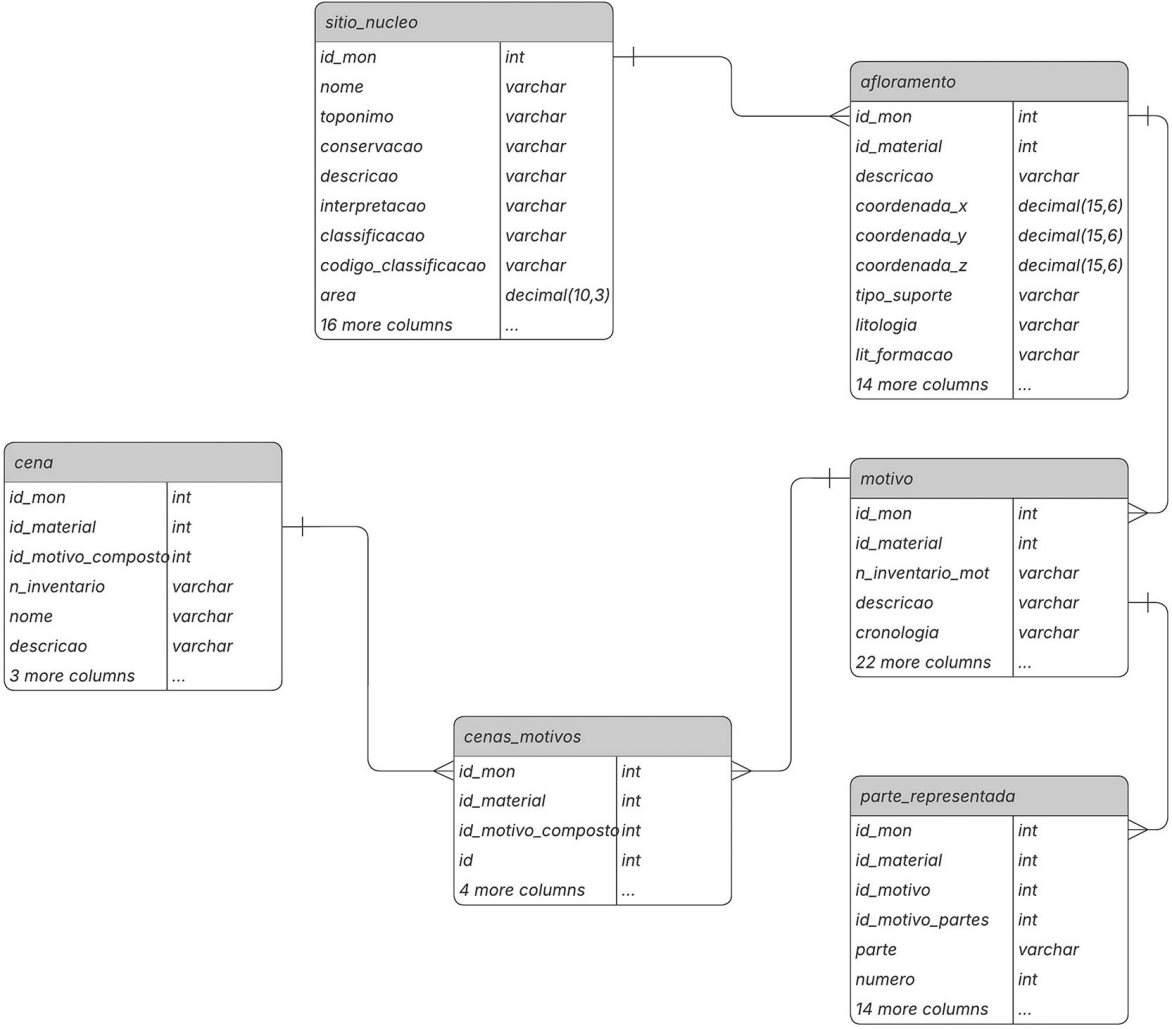
A view of the 2ArchIS entity-relationship model.

Data registration for the RARAA project was conducted using the 2ArchIS information system (see Figure 1 for a portion of its data model and the complementary data ^54^ for information on the tables' attributes), developed at the University of Minho's Archaeology Unit. This data concerns the excavated sites, the outcrops with engraved rock art, the motifs discovered, their parts, and the scenes represented. Data access was facilitated by creating a back-office application in PHP and HTML, running on a web platform and usable both in the field and in the office. All data recorded in 2ArchIS was also deposited in a repository with persistent identifiers, dataRepositóriUM, ensuring their preservation and dissemination and making them locatable and accessible.

The modular structure of the 2ArchIS information system integrates and interconnects modules for characterizing archaeological sites, stratigraphy, excavation materials, geological and geographical contexts, and rock art. We will present the information system modules, highlighting those associated with recording rocks and rock art motifs.

Archaeological Site: The archaeological site characterization module (sitio_nucleo) records the site's location, toponymy, and typology data. However, additional information is essential to associate with the site, such as its geographical, geological, hydrographic context, or land use, as these contextual data are crucial for better understanding the site and the criteria for choosing its location. Thus, we have specific tables for recording this data, namely sitio_hidrografia, sitio_contexto_geog, sitio_contexto_geol, sitio_uso_sol. Sites may also have occupation over time, meaning they are associated with various chronologies (table sitio_rela_percultural) and have multiple configurations and uses during these periods of occupation (sitio_rela_tipo). This information results from studies derived from archaeological excavations, whose material records are stored in the sitio_rela_materiais table. This data, integrated into the 2ArchIS information system, provides a more comprehensive framework for rock art.

Rocky Outcrops: This module characterizes the existing rocky outcrops at the site that support rock art. Detailed information includes the type of outcrop, such as location, size, lithology (shale, granite, others), surface morphology (flat, concave, or convex), surface appearance (fractures, nodules, polishing, etc.), and conservation status.

Motifs: On the surface of the outcrop, motifs can be identified, which can be of various types: figurative, geometric, and alphanumeric. Details recorded for each motif cover inventory number, chronology, engraving techniques, style, typology, and other characteristics. The module highlights the diversity of motifs, ranging from isolated figures to narrative scenes.

Represented Parts in motifs: The motifs characterized in the Motif module are not always complete, and even when they are, they may present unique characteristics of the design of each component, such as the shape of the head, the number and shape of the limbs represented, in this case of anthropomorphs or zoomorphs, or the shape and type of each weapon component. Therefore, in this module, we characterize each represented part of the motif, including its typology, shape, position, engraving technique, and associated measurements.

Scenes: Not all motifs stand alone; some form scenes, interacting with each other in a narrative context. The Scene module is designed to associate motifs that create these narrative compositions.

Documentation: In this module, we emphasize the system's ability to associate all entities in the 2ArchIS system with bibliographic references, photographs, drawings, cartography, and 3D models. This comprehensive documentation enriches the archaeological data and provides a more holistic understanding of the findings.

Portions of the attributes of the 2ArchIS tables are available as complementary data. ^54^ Specifically, these attributes relate to entities directly relevant to this work, such as Archaeological Site, Outcrop, Motif, Represented Part, and Scene. We highlight the case of motifs and represented parts, which share some common attributes and descriptors. A motif characterizes the representation of a figure, whether geometric, letters and numbers, or figures of people or animals, through a set of lines engraved on the rock surface. However, this engraving, done by incision using a tool, which can be a fragment of stone or metal, is not always uniform throughout the entire motif, depending on the force and/or inclination applied by the tool used for engraving. Thus, a motif may exhibit varying line depths and thicknesses. ^55^ For example, we have motifs in which the head of the anthropomorph is drawn with a deeper incision and even reinforced by more than one line, whereas the same does not occur in the representation of the torso and limbs. Therefore, while the motifs record the general characteristics of the figure, namely in the attributes of technique and variant technique, for the represented parts, we specify the same attributes to characterize the features of the rock carvings in that specific part.

The transformation of this specialized data structure into Linked Open Data presents unique challenges that require a tailored approach, which we address through our methodological framework.

## Methodological procedures

This work uses archaeological data previously collected in a relational database, developed within the RARAA project and implemented in the 2ArchIS information system. The contribution of this article lies in the semantic modeling, transformation workflow, and publication of this data as Open Data (LOD), along with the development of reusable semantic artifacts and software tools that enable FAIR access, interoperability, and reuse of rock art documentation.

To support the development of the application profile, we used the DCTAP, which provides a tabular structure for defining application profiles in a clear and understandable format. ^12^ It defines how the data should be used and what to expect from it. The current vocabulary version comprises 12 terms ^56^ used to name the DCTAP columns. The number of columns can be increased or decreased based on users' needs. Adding columns means adding terms, which should be defined for clarity. Clear definitions are essential to ensure that data can be reused effectively in open environments.

Using the Entity-Relationship (E-R) model ( Figure 1) as a basis, we first identified the shapes to include in our DCTAP. It is worth highlighting that a shape can be related to one or more entities, given that in Linked Data the properties (attributes in the E-R model) can be repeated (cardinality >1), i.e., there are no restrictions on m:n relationships. This means that entities in the E-R model may be partially or fully absorbed by others, resulting in a single shape. Another situation may arise in which an entity gives rise not to a shape but to a controlled vocabulary.

Still on the DCTAP, we replaced the propertyLabel column with an attribute column to indicate each attribute present in the E-R model. Then we listed the attributes associated with each shape. Next, for each attribute, we looked for a property with the appropriate semantics that could represent it without restrictions that would prevent its use, especially regarding domain and range. We started by looking at the ARIADNE and CIDOC CRM ontologies. Finding the property, we moved on to the following attribute. Not finding it, we turned to other schemes such as DC Terms, DBpedia, or Schema.org. Sometimes, searching in LOV was necessary. When DBpedia dbp properties had limited information, to ensure correct semantics, we created specific properties and linked them to the corresponding dbp terms via rdfs:subPropertyOf. To relate the shapes, we also sought specific properties that would allow us to express the semantics of those relationships.

We sometimes could not find properties with the appropriate semantics, domains, and ranges that suited our needs. In these cases, we had to create new properties. The same happened with some classes. The lists of values associated with the attributes in the 2ArchIS database were collected, yielding a controlled vocabulary that we encoded in SKOS. New properties and classes have been encoded in RDFS. We related these properties and classes to existing ones (through rdfs:subPropertyOf or rdfs:subClassOf) to get their semantics whenever possible.

The classes and properties vocabulary, encoded in RDFS, and the controlled vocabulary for values, encoded in SKOS, were placed in the dataRepositóriUM to obtain a DOI for each. These DOIs persistently identify the respective vocabularies. The RDFS and SKOS files were then modified to include these identifiers, generating new versions uploaded to the same record in the dataRepositóriUM. The DCTAP was also placed in the dataRepositóriUM.

To implement the application profile, we also used dataRepositóriUM to store data for each motif and obtain a persistent identifier (a DOI) for each motif. Only motifs have a DOI, so other resources have internal identifiers and, when applicable, are described in relation to the motifs. For example, scenes and outcrops relate to motifs using the ao:has_part property, and represented parts relate to motifs using the ao:is_part_of property. This option is duly represented in the DCTAP.

After completing the DCTAP and obtaining DOIs for all the motifs, it was time to export the data from the 2ArchIs database into XML files, then transform them to RDF/XML using OpenRefine, as prepared using the DCTAP. The data transformation was an iterative process that required some adjustments until the data was correctly transformed. The data was then validated using the ShEx schema. At first, there were some validation issues that, after analysis, revealed minor typos in the shapes but not in the data, which were promptly corrected. To fully comply with the FAIR principles, we also included a CC BY-SA [20] license in all RDF files.

To house the data and create the RARAA repository, we used GraphDB [21]. We also put the RDFS and SKOS vocabularies there to facilitate the queries. The triplestore can be interrogated via a SPARQL endpoint. The data was also made available as RDF/XML and Turtle files at the dataRepositóriUM.

## Results

### Selected namespace schemas

The used namespace schemas are shown in Table 1. The “raraa” prefix refers to the namespace we created for this project. The corresponding RDFS file is stored in the dataRepositóriUM so a DOI identifies it.

**Table 1.** Used namespace schemas.

Prefix	URL
ao	https://ariadne-infrastructure.eu/aocat/
cidoc	http://www.cidoc-crm.org/cidoc-crm/
dbo	https://dbpedia.org/ontology/
dbp	http://dbpedia.org/property/
raraa	https://doi.org/10.34622/datarepositorium/VVCF9M#

### RARAA properties and classes

Adhering to the best practices of the Semantic Web community, to maximize semantic interoperability, we tried to reuse existing properties and classes as much as possible, prioritizing standardized or widely used namespace schemas. The semantics, ranges, and domains of existing properties were verified for compatibility. However, despite our best efforts, we were unable to do so in some cases, leading us to create 29 new properties ( Table 2) [22]. These properties were related to existing properties to get their semantics whenever possible ( Table 3). Also, in this case, the semantics, domain, and range of existing properties were considered for compatibility. The RDFS code for these new properties can be found in the RARAA namespace URI, ^57^ as shown in Table 1.

**Table 2.** 2ArchIS attributes x RARAA namespace properties.

E-R Diagram		RARAA namespace		Definition
E-R Entity	Attribute	DCTAP shape	Property	
sitio_nucleo		Site		
	acessos		raraa:accessDescription	A site's access description, e.g., access to the site via a steep slope, sometimes covered with loose stones and occasional vegetation.
	tipo_acesso		raraa: accessType	A site's access type, e.g., public, private, or restricted.
	interpretacao		raraa:interpretation	Site interpretation.
	classificacao		raraa: siteClassification	The classification assigned to the site, such as national monument or a special protection area.
	nome		raraa:siteName	The site name.
	cod_classificacao		raraa:siteNationalCode	The site's national code issued by a national authority.
motivo		Motif		
	animacao		raraa:animationTechnique	The animation technique used to create a dynamic visual effect that suggests movement.
	grupo		raraa:graphicalGroupType	The group classifies the motifs into three categories based on their visual and symbolic characteristics: figurative, geometric, and alphanumeric.
	sub_tipo		raraa:graphicalSubType	The subtype provides a more detailed classification within the broader categories defined in group. For example, within the figurative group, subtypes include anthropomorphic, zoomorphic, and weapons.
	tipo		raraa:graphicalType	The type offers a more detailed classification within each subtype; for example, we can identify types such as equine, deer, or aurochs within the zoomorphic subtype.
	motivo_figura		raraa: completeFigure	Indicates whether the figure is complete or not.
	invertida		raraa:invertedFigure	Indicates whether the figure is inverted or not.
	orientacao		raraa:partOrientation	Orientation of motifs in degrees, e.g., 20° or 45°.
	patine		raraa:patina	Indicates whether the motif's trace has patina or not.
	perfil		raraa:perspective	Indicates the motif's perspective, which can be either frontal or absolute profile.
	relacao_suporte		raraa:relationWithRock	Indicates how the motif utilizes the support, such as fissures or inclusions.
	tecnica_variante		raraa:variantTechnique	Indicates the type of incision used in engraving the motifs. Examples include simple incision, repeated incision, abrasion, and others.
afloramento		Outcrop		
	direcao		raraa:direction	General orientation of the rock outcrop measured in degrees, for example, 30°N or 25°E.
	inclinacao		raraa:inclination	The inclination of the outcrop panel measured in degrees, for example, 87°NW.
	estratigrafia_inclinacao		raraa:inclinationStratigraphy	The stratigraphic inclination.
	lit_formacao		raraa:lithologyFormation	Name of the lithological formation, such as Desejosa or Pinhão.
	litologia		raraa:lithology	Lithology type, e.g., shale or granite.
	altura		raraa:maxHeight	Maximum height of the outcrop.
	comprimento		raraa:maxLength	Maximum length of the outcrop.
	termo		raraa:modificationTerm	Indicates whether the panel has the term “alteration” or not.
	asp'sup		raraa: surfaceAppearance	Characterization of the surface appearance, which can be, for example, smooth or heavily fractured.
	morfologia_sup		raraa:surfaceMorphology	The surface morphology, which can be flat or concave, for example.
parte_representada		Represented Part		
	parte		raraa:representedPart	Part of the motif represented, such as the head or limbs.
cena		Scene		
	nome		raraa:sceneName	The name given to the scene.

**Table 3.** RARAA namespace properties, their rdfs:subPropertyOf relations with existing properties, and their expected types of values.

RARAA property	rdfs:subPropertyOf	Expected types of values
raraa:accessDescription	ao:has_description	xsd:string
raraa:accessType	dct:type	xsd:string
raraa:animationTechnique	dbo:technique	xsd:string
raraa:direction	dbp:direction	xsd:string
raraa:graphicalGroupType	ao:has_type	IRI [24] (term from a controlled vocabulary)
raraa:graphicalSubType	ao:has_type	IRI (term from a controlled vocabulary)
raraa:graphicalType	ao:has_type	IRI (term from a controlled vocabulary)
raraa:completeFigure	ao:has_description	xsd:string
raraa:invertedFigure	ao:has_description	xsd:string
raraa:inclination	dbp:inclination	xsd:string
raraa:inclinationStratigraphy	dbp:inclination	xsd:string
raraa:interpretation	ao:has_description	xsd:string
raraa:lithologyFormation	N/A	IRI (term from a controlled vocabulary)
raraa:lithology	dbo:material	IRI (term from a controlled vocabulary)
raraa:maxHeight	dbo:height	xsd:decimal (centimeters)
raraa:maxLength	dbo:length	xsd:decimal (centimeters)
raraa:modificationTerm	ao:has_description	xsd:string
raraa:partOrientation	dbp:direction	xsd:string
raraa:patina	N/A	IRI (term from a controlled vocabulary)
raraa:perspective	N/A	xsd:string
raraa:relationWithRock	ao:has_description	xsd:string
raraa:representedPart	dbo:pictureDescription	IRI (term from a controlled vocabulary)
raraa:sceneName	ao:has_title	xsd:string
raraa:siteClassification	dbo:classification	xsd:string
raraa:siteName	ao:has_place_name	xsd:string
raraa:siteNationalCode	ao:has_identifier	xsd:string
raraa:surfaceAppearance	N/A	IRI (term from a controlled vocabulary)
raraa:surfaceMorphology	N/A	IRI (term from a controlled vocabulary)
raraa:variantTechnique	N/A	IRI (term from a controlled vocabulary)

The rdfs:subPropertyOf relationships between RARAA properties and existing properties may be found in Table 3. The third column in this table shows the expected types of values. We did not constrain the domain or range of the properties we created so that they can be used in other situations (provided the semantics is adequate).

The RARAA namespace also contains three classes: raraa: Motif, raraa: RepresentedPart, and raraa: Scene. All three are subclasses of cidoc: E25_Human-Made_Feature (via rdfs:subClassOf property). Creating these classes was necessary to distinguish the resources by their class using the rdf:type property.

### Controlled Vocabularies

A SKOS-encoded controlled vocabulary was created based on the 2ArchIS lists of values as presented by Botica, Luís, and Silva ^8^ and available as complementary data, which provides an English translation of the original values. ^54^ Each term has an identifier and at least one prefLabel. We encoded and made available one SKOS vocabulary containing 297 concepts with English, Portuguese, and Spanish labels. The vocabulary is organized as a tree structure with six levels. The vocabulary structure reflects part of the shapes and properties with which the values/concepts are used. Leaf concepts were reused as much as possible within the vocabulary.

**Figure 2.**
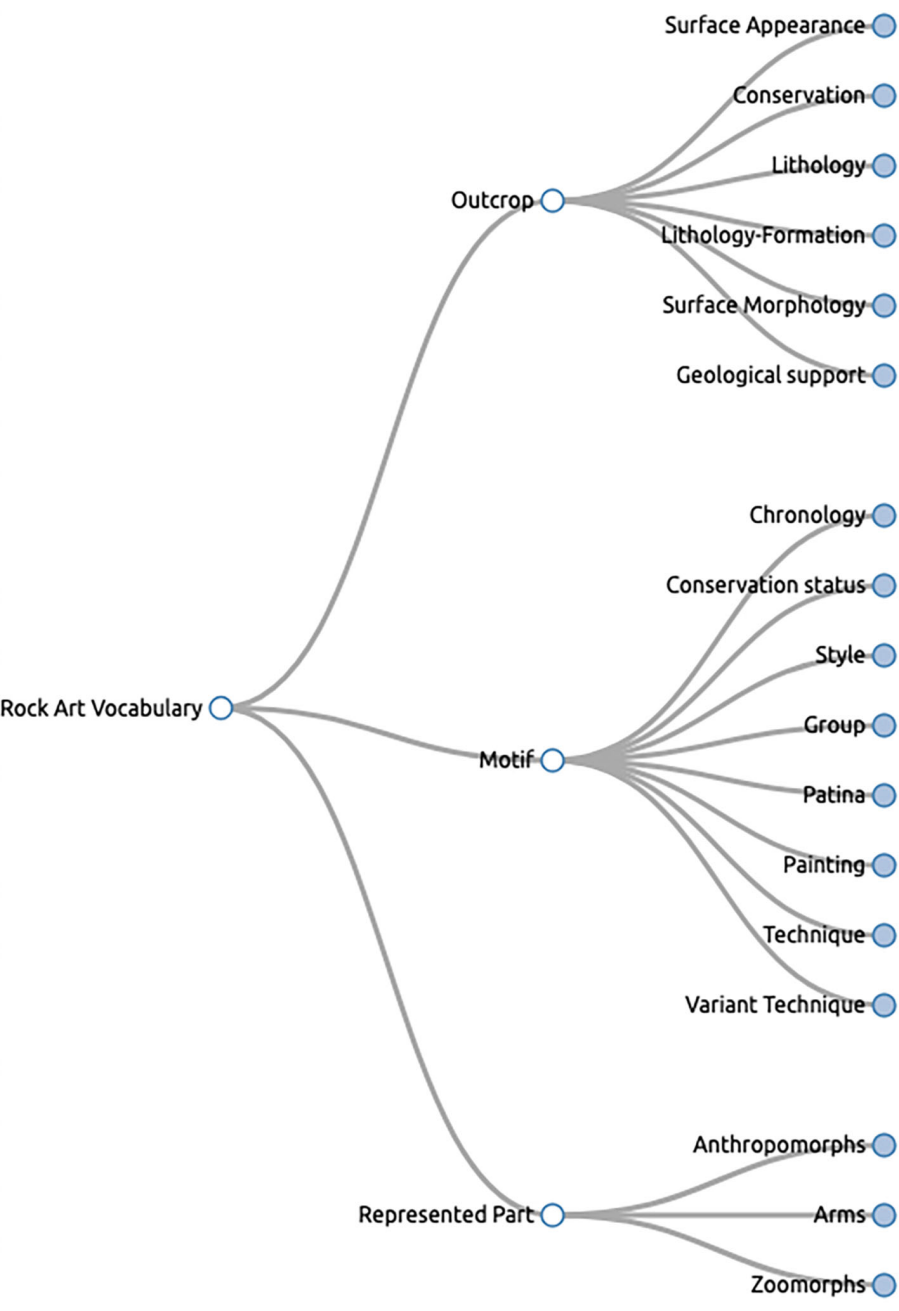
The first three levels of the RARAA values vocabulary for rock art (tool: SKOS Play !).

This vocabulary is available online. ^58^ See Figure 2 for a screenshot of the first three vocabulary levels as depicted by SKOS Play ! [23]

### Dublin core tabular application profile

We considered five shapes in the DCTAP: Motif, Scene, Represented Part, Site, and Outcrop. The shapes are related via ao:is_part_of and ao:has_part properties. As mentioned earlier, only motifs have a DOI, so scenes, represented parts, and outcrops point to motifs, and motifs have no outgoing links to other shapes’ resources. This choice preserves the integrity of the relationships since the motifs’ identifiers are persistent.

**Figure 3.**
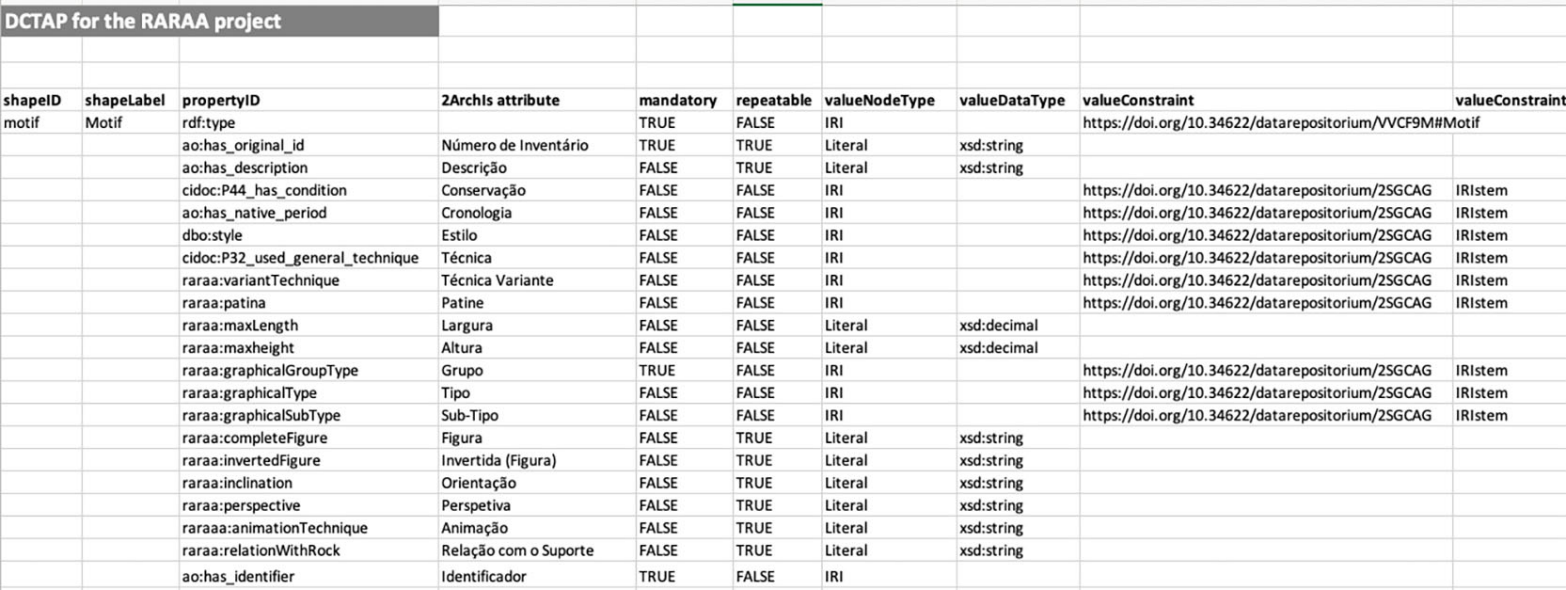
Part of the shape Motif of the RARAA project DCTAP.

The instructions in the DCTAP Primer were strictly followed. Since it was not mandatory, we replaced the propertyLabel column with the attribute column for better reference when designing the DCTAP. In properties whose values should come from a controlled vocabulary, we use the value constraint type IRIstem. We used the rdf:type property with a value constraint for the corresponding class in each shape. For example, we used the value constraint raraa: Motif for the Motif shape. Figure 3 shows the DCTAP for the shape Motif. The complete DCTAP for the RARAA project is available online. ^59^

### RDF files

**Figure 4.**
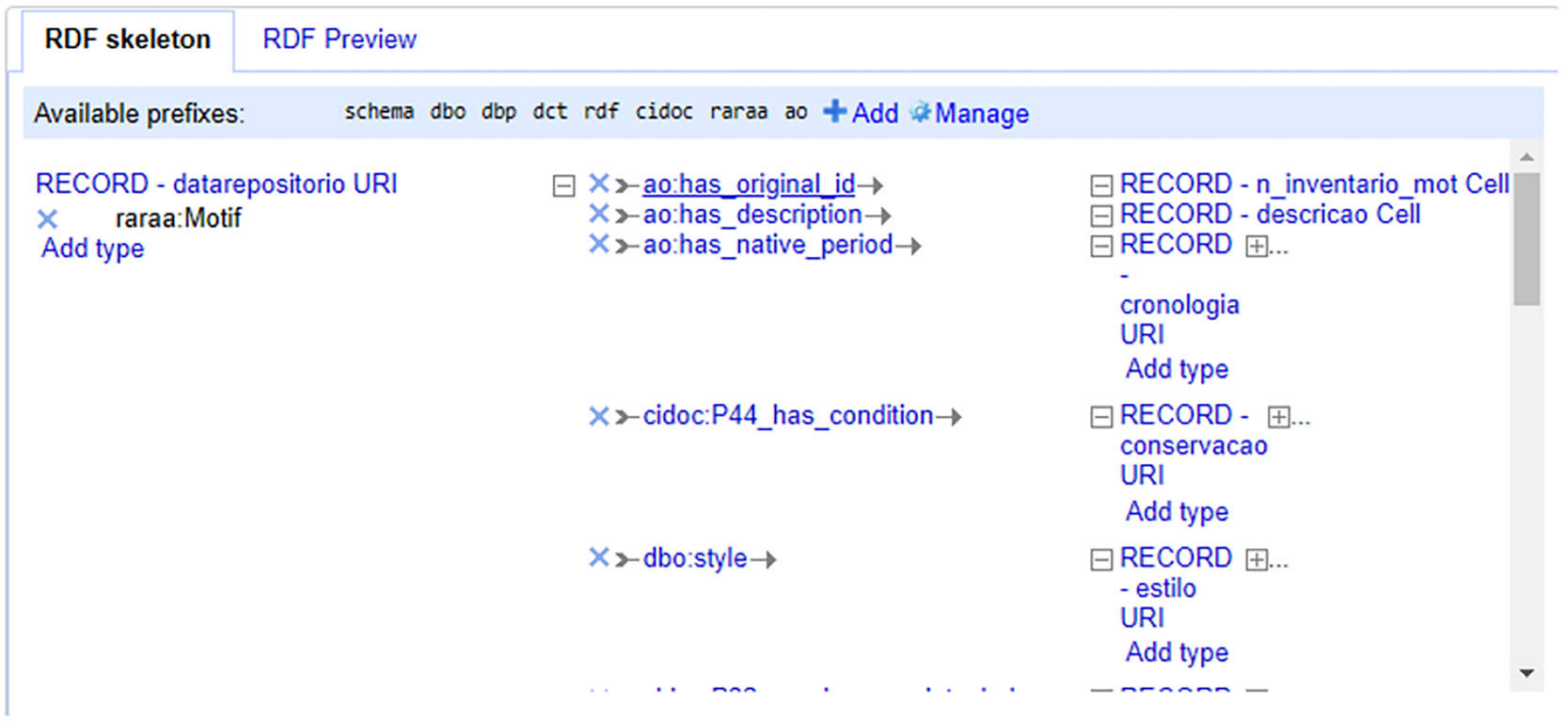
The first part of the RDF Skeleton for the Motivo (Motif ) table.

We automatically generated RDF files using OpenRefine. Having, on the one hand, the data in XML format as exported from 2ArchIS and, on the other, the DCTAP and the links of the namespace schemas and controlled vocabularies, we created RDF skeletons. In these RDF skeletons, we defined the prefix table. Then, we chose the properties using the prefix of the respective namespace, followed by information about the corresponding database column. This process specifies the transformations from entities' attributes to RDF properties, ensuring the data conforms to the project's requirements. All files regarding each table had their RDF skeleton defined. Using these RDF skeletons allowed OpenRefine to export RDF files with the defined structure so that we could later, after validation, upload the data to the GraphDB triplestore. See Figure 4 for part of the RDF skeleton for the table Motivo (Motif ).

**Figure 5.**
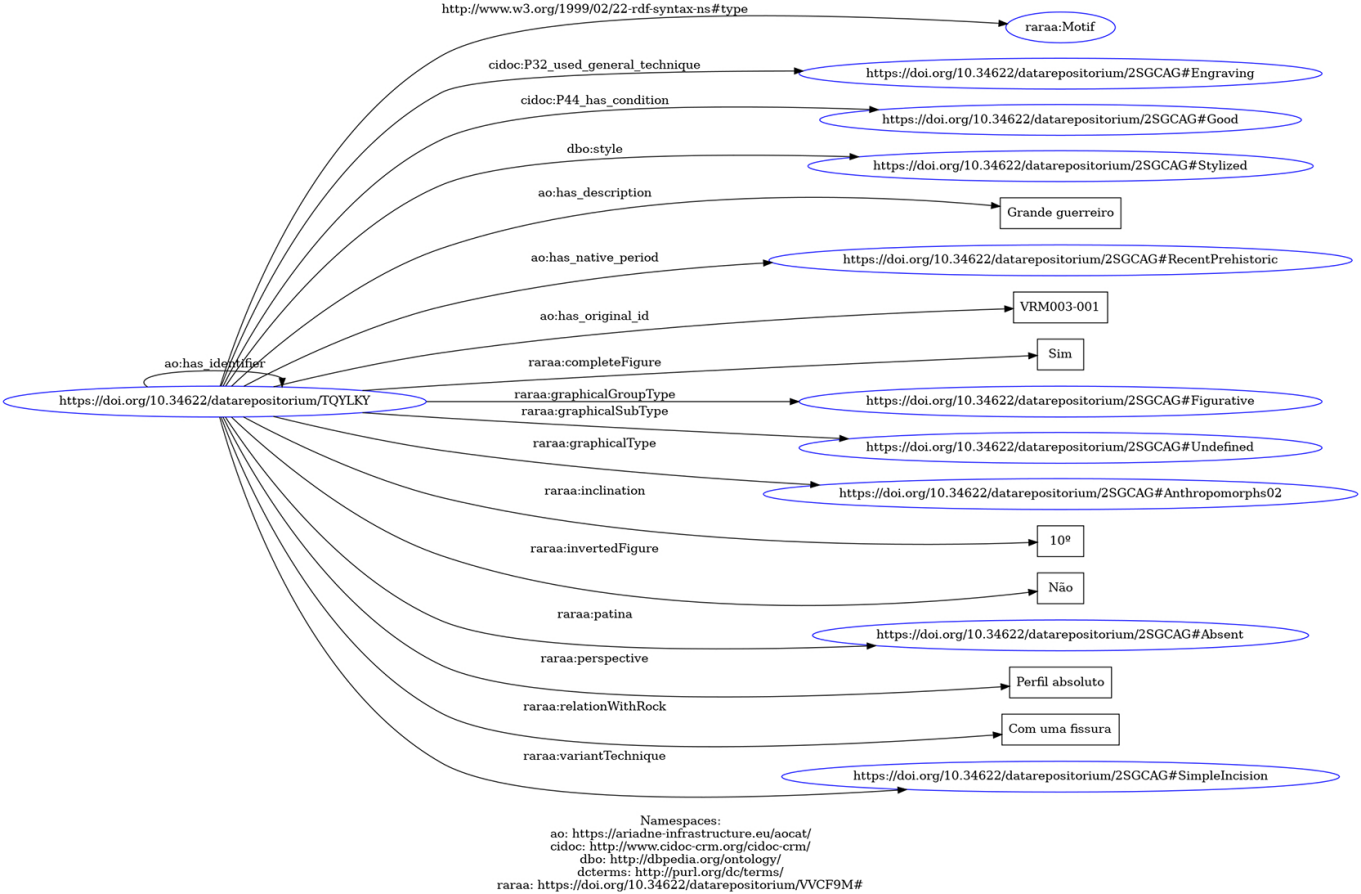
RDF graph for the "VRM003-001" motif described as "Grande guerreiro" (tool: RDF Grapher).

The RDF files are available online. ^60^ For example, Figure 5 shows the RDF graph for the "VRM003-001" motif, described as “Grande guerreiro” ("Great warrior"), generated by the RDF Grapher [25].

### Shape expressions

A ShEx schema is “a collection of shape expressions that describe an RDF graph”. ^13^ Our ShEx schema has expressions for five shapes, one for each DCTAP shape, with two purposes: 1) to validate the data generated by OpenRefine; and 2) to provide the rock art community with shapes that allow them to create and validate data using our application profile. In this way, we are creating conditions for others to provide data that can be easily added and linked to ours to advance research into rock art.

**Figure 6.**
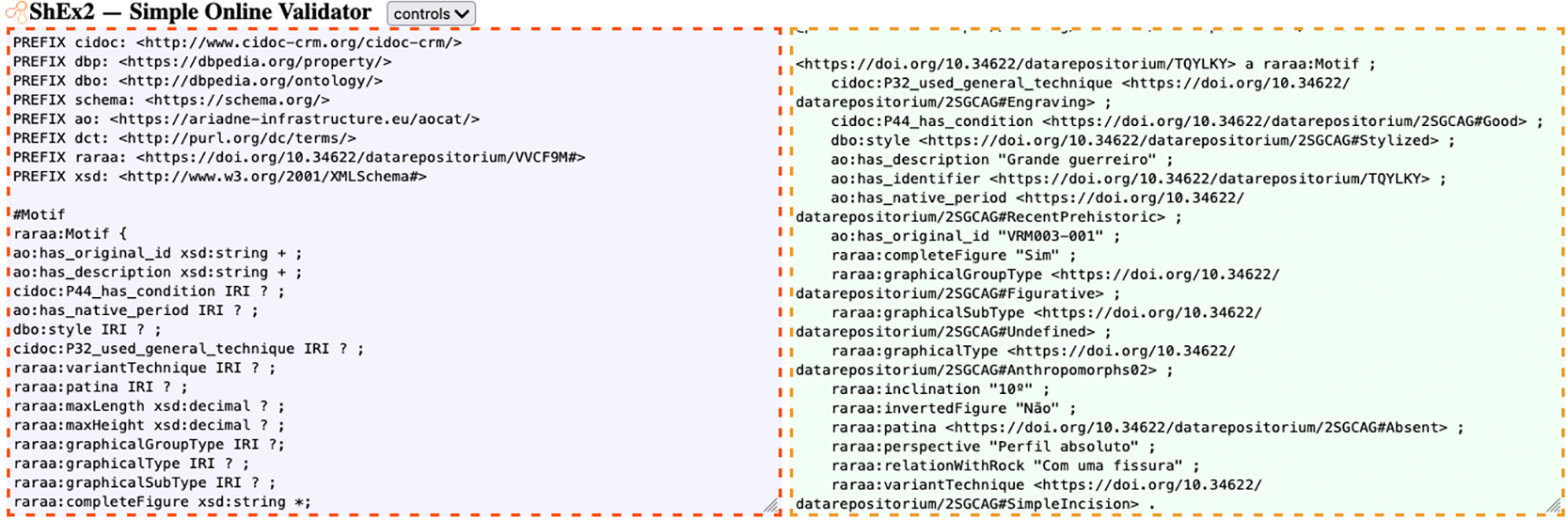
Screenshot of ShEx validation for a motif (tool: ShEx2). ^1^

As for validating the structure of our RDF code, the shape expressions ensured validation of each triple data type and verified that the properties defined in each shape were adequately used. In this process, we used an online tool [26]. We manually developed ShEx code and utilized RDF code exported from OpenRefine in Turtle format (Terse RDF Triple Language). Figure 6 shows an example of ShEx validation of a motif’s description.

The validation was straightforward once we aligned the shape expressions with the DCTAP. Since the shape expressions specified that most properties are optional, the presence of objects lacking specific properties, often due to null values, did not pose an issue. Another contributing factor to the data's validity is that the data types were strictly aligned with those defined in the shapes, as documented in the ShEx schema available online. ^61^

### The triplestore

The RARAA triplestore was created using GraphDB. The new namespace schema, controlled vocabulary, and RDF files were uploaded to the triplestore, resulting in 6.085 explicit RDF statements. The data is available through a SPARQL endpoint ^62^ and in dataRepositóriUM. ^57, 58, 60^

**Figure 7.**
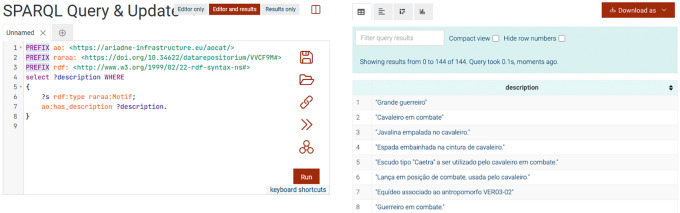
Part of the results of the SPARQL query to list all the descriptions of all motifs.

Figure 7 shows a screenshot of the results of a SPARQL query to list all motifs' descriptions. As with other triplestores, its practical use depends on understanding the application profile, its shapes, and the relationships between them. To help maximize the potential for reusing this data, an upcoming article will detail and provide more examples of potentially useful queries. Because these data were designed to maximize semantic interoperability, they can be easily integrated with other datasets from the same or different domains, enabling cross-domain analyses and comparative studies.

### Files made available for download

The following files were made openly available for use and reuse:

• DCTAP; Distribution in a.XLSX Excel file and corresponding distributions in three.CSV files: the first with the metadata, the second with the DCTAP, and the third with information about the terms of the RARAA namespace - https://doi.org/10.34622/datarepositorium/IWOZHJ ^59^
• RDF schema; Distribution in RDF/XML and Turtle (TTL) of the RARAA namespace RDF Schema - https://doi.org/10.34622/datarepositorium/VVCF9M ^57^
• Controlled vocabulary; Distribution in RDF/XML and TTL of the SKOS file with the controlled vocabulary for values - https://doi.org/10.34622/datarepositorium/2SGCAG ^58^
• ShEx schema; Distribution in Shape Expressions Compact format (ShExC) for the five shapes: Site, Outcrop, Scene, Motif, and Represented Part- https://doi.org/10.34622/datarepositorium/TVJMZY ^61^
• RDF files; Distributions in RDF/XML and TTL of the descriptions of 144 motifs, seven scenes, 181 represented parts, six outcrops, and one site (Vale da Vermelhosa) - https://doi.org/10.34622/datarepositorium/CQ42YJ ^60^

These openly available resources are structured and standardized to facilitate reuse, not only within rock art studies but also in other domains that require semantic interoperability, such as cultural heritage documentation, archaeological data integration, or linked data research in environmental and spatial sciences. For instance, the RDF files and controlled vocabularies could support comparative studies of symbolic systems, integration of archaeological datasets for predictive modeling, or enrichment of museum and heritage digital platforms. At the same time, the SPARQL endpoint, combined with visualization tools, enables automated querying, visualization, and analysis of spatial, temporal, or thematic patterns across diverse datasets.

## Conclusions and future work

Rock art represents a unique cultural heritage that intertwines artistic expression with its natural landscape. Through the RARAA project, we have prioritized documenting Iron Age rock art in the Côa Valley, adhering to FAIR principles to ensure our data are openly accessible and reusable for new interdisciplinary studies and interpretations.

The Semantic Web provides powerful tools for archaeology and rock art studies, enabling researchers to access, integrate, and interpret data more effectively while fostering broader collaboration. By working with semantic standards, associating detailed metadata with archaeological resources, and creating controlled vocabularies, and classes and properties vocabularies, we facilitate deeper discovery and understanding of this precious heritage.

Data interoperability enables archaeologists to connect and integrate data from different sources and formats across various disciplinary areas, such as geography and anthropology, which is fundamental to archaeological study and interpretation. Efficient and comprehensive access to and sharing of data are urgent needs in archaeology, promoting the work of researchers and fostering collaboration among researchers, projects, and institutions.

To make data from rock art studies in the Côa Valley conducted by the RARAA project available to other researchers, we created an open data repository aligned with the FAIR principles and emblematic projects in the area, including ARIADNE and ARIADNEplus. This repository is implemented in a triplestore, which can be accessed via a SPARQL endpoint. ^62^ Its creation involved several steps, from the study of the 2ArchIS database, the definition of the application profile using DCTAP, the definition of a new namespace schema (RDFS) and one controlled vocabulary schema (SKOS), the definition of a ShEx Schema, the transformation of data using OpenRefine, the validation of the data against the ShEx Schema, and, finally, uploading the data to the triplestore. All these outputs are available in dataRepositóriUM, the University of Minho's data repository. ^57– 61^ Both the data made available via triplestore and those made available via dataRepositóriUM are helpful for other archaeology or Linked Data researchers, students, data managers, and interested citizens.

### Contributions to theory and practice

This work provides three distinct contributions to the field of cultural heritage data management—methodological, technical, and theoretical.

From a methodological perspective, we explicitly describe a complete and reproducible workflow for converting specialized rock art data from relational databases into Linked Open Data, using DCTAP as the core model for developing application profiles. One of the purposes of adopting DCTAP was to assess its ability to express the various requirements that emerge during the design of application profiles. In our case, it successfully addressed almost all our needs throughout the process. Nevertheless, we could not express the intention of using part of a vocabulary (a branch, for example) as a value constraint. The possibility of using an IRIstem, combined with an adequate design of SKOS vocabularies, could overcome this expression difficulty. However, it would be more interesting to use a more expeditious and transparent way without resorting to subterfuges like that. Therefore, as a theoretical contribution, we can state that DCTAP effectively fulfilled its purpose, while also identifying a potential improvement: the ability to indicate one or more branches of a vocabulary as value constraints.

The University of Minho's open data repository was used as a key component to assign persistent identifiers to both motifs and developed artifacts, while simultaneously ensuring the long-term digital preservation of these resources. Documenting these specific steps within the overall project workflow is crucial, particularly for low-budget initiatives that lack the internal capacity to ensure identifier persistence and long-term preservation autonomously.

As technical contributions, we provide a set of reusable artifacts that other researchers and developers can adopt in their own projects involving either open or closed data: an application profile for the description of rock art expressed using DCTAP; a new schema of properties encoded in RDFS; a controlled vocabulary encoded in SKOS; a ShEx schema corresponding to the application profile shapes; and RDF files relating to rock art data stored in the 2ArchIS database. All these resources are openly available for use and reuse in dataRepositóriUM, alongside a triplestore accessible via its SPARQL endpoint. The developed vocabularies, being semantically interoperable, can be reused in other rock art or archaeology projects. The published LOD can also be integrated with data from different domains, fostering new perspectives and insights.

Our work also demonstrates how to balance domain-specific requirements through customized properties while maintaining cross-domain interoperability by aligning with major semantic frameworks, including CIDOC CRM, Ariadne, DBpedia, and schema.org. The online availability of the property, class, and value vocabularies supports their discussion, refinement, and potential integration with existing or future vocabularies, thereby contributing to both practice and theory in rock art knowledge organization. This process enhances the reusability of rock art data in new projects and interpretations, facilitating broader analyses at national and international scales.

Overall, this approach provides a sustainable model for other specialized archaeological subdisciplines seeking to publish their data as Linked Open Data, combining methodological rigor, semantic alignment, and practical reusability.

### Limitations and future work

Like all research projects, this one has limitations, summarized below. The RARAA project brought together specialists from different fields, archaeologists responsible for identifying and characterizing the motifs, and information technology experts in charge of digital surveys and Linked Open Data modeling. With the end of the project’s funding, continuing this interdisciplinary work requires the rock art research team to create new funding opportunities or to rely on the voluntary contribution of data specialists to maintain and expand the Linked Open Data component.

The first limitation is that the source data (the 2ArchIS database) was not originally designed for data sharing and primarily reflects the perspective of UAUM researchers. The structure of the source data, such as the attributes used, the controlled vocabularies and their organization, has a substantial impact on the design of the data to be published as Linked Data. Aligning these data with existing namespace schemas and controlled vocabularies is challenging, especially if those schemas and vocabularies already have predefined structures or explicit constraints that limit the reuse of their terms. For example, some of the properties we use must be able to be related to terms from our controlled vocabulary. This implied that certain properties from relevant schemas could not be used due to their range constraints. Our alignment efforts focused mainly on the ARIADNE and ARIADNEplus projects. However, these projects have not yet considered rock art-specific data, focusing more on archaeological excavation datasets, such as stratigraphy and materials. Consequently, several of the properties and classes we required were absent. To address this, we used more general vocabularies and ontologies, such as DBpedia, Schema.org, and Dublin Core, and even created new properties. Our application profile can therefore serve as a starting point for other projects that include or will include data on rock art, including ARIADNEplus.

In this project, we did not establish any connection between the 2ArchIS database and the triplestore. We first exported the data from 2ArchIS to XML and then imported it into OpenRefine, where we transformed it into RDF. As the 2ArchIS database is expected to continue operating and remain the primary source of data for the triplestore, it is crucial that we implement, in the future, a mechanism that performs these operations automatically or, at least, semi-automatically, based on the ShEx expressions we developed and made available.

Currently, we only make the data available via the SPARQL endpoint, which can be difficult for users unfamiliar with SPARQL. This limitation may jeopardize timely access to data for some researchers, underscoring the need to develop a user-friendly interface that enables non-technical users to access the data. This interface, expected to be available soon, will replicate some of 2ArchIS's functionalities, allowing external researchers to query the data like UAUM archaeologists do. Exporting and downloading data in open formats, such as CSV, should also be possible in the future. Providing such features may encourage other archaeological research units to adopt similar practices, mainly Portuguese, Ibero-speaking, and European. Other actions may include developing tutorials or training materials and sessions, and making them available online.

Currently, our triplestore provides access only to the data, the schema, and the controlled vocabulary we created. It would be interesting in the future to enrich these data with data from other sources, including other institutions with rock art or related themes. It may, for instance, include geographical information in the form of GeoNames or TGN IRIs. It could also be enriched with specific ARIADNE, DBpedia, or Wikidata data. In this case, the interface for non-technical humans should allow querying all data to discover new relationships and insights. Our data can also contribute to other data stores, such as Wikidata – efforts in this direction are needed. Future work may also include defining mappings between our controlled vocabulary and other vocabularies or ontologies, such as MAO/TAO, to enhance semantic interoperability.

Finally, new opportunities will emerge from integrating our data into the Social Sciences and Humanities Open marketplace and the Common European Data Space for Cultural Heritage. With our Linked Data we have already met several technical requirements. Still, others will need to be met, such as the use of the Data Catalogue Vocabulary (DCAT), and other operational and institutional requirements will need to be addressed in future work.

## Ethics and consent

Ethical approval and consent were not required.

## Data Availability

The data used in this study originates from the 2ArchIS relational database developed at the University of Minho within the scope of the RARAA project. No new primary archaeological data were generated for this work. All datasets and semantic artifacts produced and used in the context of this study are openly available through the University of Minho’s institutional data repository, dataRepositóriUM. These include the complementary original data, the Linked Open Data representations of rock art motifs (in RDF/XML and Turtle formats), the application profile defined using DCTAP, the namespace schema encoded in RDFS, the controlled vocabularies encoded in SKOS, and the ShEx schema used for data validation. Each artifact is individually identified with a persistent Digital Object Identifier (DOI), ensuring long-term preservation, citability, and unambiguous reference. The available data last versions are the following:
1. dataRepositóriUM. RARAA DCTAP. https://doi.org/10.34622/datarepositorium/IWOZHJ ^59^
•
DCTAP_RARAA_FinalV3_1.tab -.XLSX file with the RARAA Application Profile (DCTAP). •
DCTAP_Metadata_V3_1.tab-.CSV file with the Application Profile catalog metadata. •
DCTAP_RARAA_FinalV3_1CSV.tab-.CSV file with the RARAA Application Profile (DCTAP). •
Terms_RARAA_NamespaceV3_1.tab-.CSV file with information about the terms of the RARAA namespace. Data is available under the terms of the CC0 - “Public Domain Dedication” - https://creativecommons.org/public-domain/cc0/. 2. dataRepositóriUM. RARAA Properties and Classes Vocabulary for Rock Art. https://doi.org/10.34622/datarepositorium/VVCF9M ^57^
•
RARAA_RockArtPropertiesVocabularyV2_1.rdf – RDF/XML file with RARAA namespace RDF Schema. •
RARAA_RockArtPropertiesVocabularyV2_1.ttl - Turtle file RARAA namespace RDF Schema. Data is available under the terms of the CC0 - “Public Domain Dedication” - https://creativecommons.org/public-domain/cc0/. 3. dataRepositóriUM. RARAA Values Vocabulary for Rock Art. https://doi.org/10.34622/datarepositorium/2SGCAG ^58^
•
RARAA_RockArtValuesVocabularyV2_1skos.rdf - RDF/XML file with SKOS RARAA Values Vocabulary for Rock Art. •
RARAA_RockArtValuesVocabularyV2_1skos.ttl - Turtle file SKOS RARAA Values Vocabulary for Rock Art. Data is available under the terms of the CC-BY-4.0 - “Creative Commons Attribution” - https://creativecommons.org/licenses/by/4.0/. 4. dataRepositóriUM. RARAA Shape Expressions. https://doi.org/10.34622/datarepositorium/TVJMZY ^61^
• RARAAShexFileV2.shex – Shape Expressions Compact format (ShExC) file for the five shapes: Site, Outcrop, Scene, Motif, and Represented Part. • ShEx_ValidationV2.jpg - Screenshot with a validation example. Data is available under the terms of the CC0 - “Public Domain Dedication” - https://creativecommons.org/public-domain/cc0/. 5. dataRepositóriUM. RARAA Rock Art Data. https://doi.org/10.34622/datarepositorium/CQ42YJ ^60^
• motifV2_1.rdf - RDF/XML data for motifs. • motifV2_1.ttl - Turtle data for motifs. • outcropV2_1.rdf - RDF/XML data for outcrops. • outcropV2_1.ttl - Turtle data for outcrops. • representedPartV2_1.rdf - RDF/XML data for represented parts. • representedPartV2_1.ttl - Turtle data for represented parts. • sceneV2_1.rdf - RDF/XML data for scenes. • sceneV2_1.ttl - Turtle data for scenes. • siteV1_1.rdf - RDF/XML data for sites. • siteV1_1.ttl - Turtle data for sites. Data is available under the terms of the CC-BY-4.0 - “Creative Commons Attribution” - https://creativecommons.org/licenses/by/4.0/. 6. dataRepositóriUM. 2ArchIS tables attributes values. https://doi.org/10.34622/datarepositorium/XPSP7L ^54^
•

00_ReadMe_file.txt – ReadMe texto file with the description of the dataset. • 01_2ArchIS tables and attributes values.tab-.XLSX file with information about 2ArchIs tables, attributes and values used within the RARAA project. • 02_2ArchIS tables and attributes values.csv - 2ArchIS enumerated values and respective tables and attributes, excluding Group, Type and Sub-type (terms translated to English). • 03_2ArchIS tables and attributes values.csv - 2ArchIS enumerated values and respective tables and attributes for Group, Type and Sub-Type (terms translated to English). • 2ArchIS tables attributes values.csv - 2ArchIS enumerated values and respective tables and attributes, excluding Group, Type and Sub-type (terms translated to English). Data is available under the terms of the CC0 - “Public Domain Dedication” - https://creativecommons.org/public-domain/cc0/.

## Footnotes

[1]
https://finto.fi/maotao/en/

[2]
https://digital-strategy.ec.europa.eu/en/policies/data-spaces

[3]
https://www.dataspace-culturalheritage.eu

[4]
https://dspace.org/

[5]
https://www.w3.org/TR/vocab-dcat-3/

[6]
https://op.europa.eu/pt/web/eu-vocabularies/dcat-ap

[7]
https://data.europa.eu

[8]
https://www.dbpedia.org/

[9]
http://dbpedia.org/resource/

[10]
http://dbpedia.org/property/

[11]
https://www.wikidata.org

[12]
https://wikimediafoundation.org/

[13]
https://creativecommons.org/publicdomain/zero/1.0/

[14]
https://schema.org/

[15]
https://www.geonames.org/

[16]
https://www.getty.edu/research/tools/vocabularies/tgn/

[17]
https://creativecommons.org/licenses/by/4.0/

[18]
https://opendatacommons.org/licenses/by/1-0/

[19]
https://lov.linkeddata.es/dataset/lov/

[20]
https://creativecommons.org/licenses/by-sa/4.0/

[21]
https://www.ontotext.com/products/graphdb/

[22] See the complementary data, for a complete list of 2ArchIS tables and respective attributes (terms translated to English) used in this project.

[23]
https://skos-play.sparna.fr/play/

[24]
https://www.ldf.fi/service/rdf-grapher

[25]
https://shex.io/webapps/shex.js/doc/shex-simple.html

[26]
https://doi.org/10.34622/datarepositorium/2SGCAG
