# Supplementary material for: Vale do Côa rock art as linked open data: Application profile, schemas, and data
Source: Open Res Eur. 2026 Jul 28;6:63. Originally published 2026 Mar 3. [Version 3] doi: 10.12688/openreseurope.21901.3 (PMC13221625; doi:10.12688/openreseurope.21901.3)
Supplement: Peer review history

# Reviewer report: Reviewer response for version 3

**DOI:** 10.21956/openreseurope.26498.r70885

**Published:** 2026-08-04

**Authors:** Diego Garate

**Affiliations:** 1 Universidad de Cantabria, Santander, Spain

I approve the changes made by the authors.

Is the study design appropriate and does the work have academic merit?

Yes

Is the work clearly and accurately presented and does it cite the current literature?

Partly

If applicable, is the statistical analysis and its interpretation appropriate?

Not applicable

Are all the source data underlying the results available to ensure full reproducibility?

Partly

Are the conclusions drawn adequately supported by the results?

Yes

Are sufficient details of methods and analysis provided to allow replication by others?

Yes

Reviewer Expertise:

NA

I confirm that I have read this submission and believe that I have an appropriate level of expertise to confirm that it is of an acceptable scientific standard.

# Reviewer report: Reviewer response for version 2

**DOI:** 10.21956/openreseurope.25768.r74077

**Published:** 2026-05-29

**Authors:** Diego Garate

**Affiliations:** 1 Universidad de Cantabria, Santander, Spain

This work presents a significant initiative in the field of archaeological data management and openness, addressing the transformation of the information contained in the 2ArchIS database into an accessible and reusable Linked Open Data (LOD) dataset.

 It is a technically sound, well-documented contribution, aligned with current trends in the openness, interoperability and digital preservation of archaeological heritage. However, beyond the quality of the technical development, the manuscript raises several issues that limit its scientific scope, and which should be addressed before considering its final indexing.

 The main issue lies in the imbalance between the technological dimension of the work and its archaeological contribution. Although the article is presented within the field of rock art and takes the Côa Valley as its case study, the discussion focuses almost exclusively on aspects related to semantic modelling, the construction of controlled vocabularies and data transformation procedures. The reader is provided with a detailed description of the computational processes developed, but there is little explanation of the implications this new model may have for archaeological research itself.

 Throughout the text, it is repeatedly stated that opening up the data will enable new insights into rock art to be generated or facilitate comparative analyses at different scales. However, these claims remain at the level of potential and are not substantiated by concrete examples. It would have been desirable to include a case study illustrating how the proposed structure allows archaeological questions to be formulated that were difficult or impossible to address using the original database. Similarly, the absence of examples of effective integration with other repositories or datasets limits the reader’s ability to assess the true scope of the proposal.

 In this regard, the manuscript also suffers from a lack of evaluation of the developed model. Formal validation using ShEx ensures the structural consistency of the data, but does not provide information on the scientific utility of the system. It would be advisable to incorporate some form of comparative evaluation to assess the model’s advantages over other existing solutions or over the original relational structure itself. Currently, the work demonstrates that the transformation is technically possible, but fails to fully demonstrate its benefits for research.

 Another aspect worthy of attention is the actual size of the published dataset. Although the introduction places the work in the context of the more than 1,200 known engraved rocks in the Côa Valley, the model is ultimately applied only to a relatively small set of records corresponding to a small part of the RARAA project. This does not invalidate the work, but it does call for a more explicit reflection on the representativeness of the sample and on the limitations this imposes on the future reuse of the data.

 From an archaeological perspective, the literature review is insufficient. The article engages extensively with the literature on ontologies, metadata and the Semantic Web, but makes scant reference to work carried out over recent decades on the digital documentation of rock art or the management of archaeological databases. Particularly striking is the scarcity of references relating to the digital documentation and analysis of rock art, as well as the absence of a more in-depth discussion of international projects that have addressed similar problems from different perspectives.

 Certain sections, particularly the introductory glossary and parts of the methodological description, are excessively lengthy in relation to the subsequent scientific discussion. The detailed listing of properties, classes and vocabularies takes up considerable space that could be partially moved to supplementary materials. Such a reorganisation would allow for the reinforcement of aspects that are currently dealt with too briefly, such as the scientific implications of the project, its potential for reuse, or its contribution to the study of rock art.

 Consequently, I believe the article is of sufficient merit to be indexed, but it requires revision to strengthen its archaeological dimension and to demonstrate more clearly the scientific contribution that this new data environment offers to the study of rock art, beyond the technological innovation itself.

Is the study design appropriate and does the work have academic merit?

Yes

Is the work clearly and accurately presented and does it cite the current literature?

Partly

If applicable, is the statistical analysis and its interpretation appropriate?

Not applicable

Are all the source data underlying the results available to ensure full reproducibility?

Partly

Are the conclusions drawn adequately supported by the results?

Yes

Are sufficient details of methods and analysis provided to allow replication by others?

Yes

Reviewer Expertise:

Rock Art

I confirm that I have read this submission and believe that I have an appropriate level of expertise to confirm that it is of an acceptable scientific standard, however I have significant reservations, as outlined above.

# Response

**Published:** 2026-06-29

**Authors:** Ana BAPTISTA

**Affiliations:** Information Systems, Universidade do Minho Centro ALGORITMI, Guimaraes, Braga, Portugal

We thank the reviewer for the careful reading of the manuscript and for the constructive comments. We are pleased that the reviewer recognizes the technical soundness of the work and its contribution to archaeological data management, interoperability, and openness. Regarding the balance between the technological and archaeological dimensions of the article, we would like to clarify that the primary objective of this contribution is methodological. The manuscript focuses on the process of transforming archaeological data from a relational database into Linked Open Data and on the development of the semantic artifacts required to support this transformation. For this reason, the emphasis is placed on the design of the application profile, vocabularies, validation mechanisms, and publication workflow rather than on archaeological interpretation. We agree that Linked Open Data environments may ultimately support new archaeological questions and comparative analyses. However, demonstrating such outcomes would require a separate research stage involving the integration of additional datasets and the formulation of specific archaeological case studies. At the current stage, the objective was to establish and document a reusable semantic framework capable of enabling such future research. We have therefore been careful throughout the manuscript to frame these possibilities as potential benefits rather than as demonstrated outcomes. Concerning the evaluation of the model, our intention was to validate the consistency and completeness of the data representation through the application profile and ShEx validation process. The article does not aim to compare alternative modeling approaches or database technologies, but rather to document a complete and reproducible workflow for publishing archaeological rock art data as Linked Open Data. We acknowledge that comparative assessments of different semantic models and their research outcomes would constitute valuable future work. With respect to the size of the published dataset, we agree that the scope should be made explicit. The manuscript now clarifies that the Linked Open Data workflow was developed and validated using the Vermelhosa dataset, corresponding to a limited number of engraved rocks within the broader RARAA project. We regard this dataset as a pilot implementation that establishes the foundations for the future incorporation of additional records from Vale de José Esteves, Meijapão, and other areas of the Côa Valley. Regarding the literature review, we acknowledge the importance of the broader literature on digital rock art documentation and archaeological information systems. However, given the specific focus of this article on semantic modeling, Linked Data publication, and interoperability, we prioritized references directly related to ontologies, metadata standards, application profiles, and archaeological Linked Open Data initiatives. We recognize that a more extensive review of digital rock art documentation projects could further enrich the archaeological context and represents a valuable avenue for future work. Finally, concerning the length of some methodological sections, we understand the reviewer’s concern. However, we intentionally provide a detailed description of the modeling decisions, vocabularies, and workflows because one of the central contributions of the paper is to offer a transparent and reproducible methodological framework that can be adopted and adapted by other researchers and institutions working with archaeological and cultural heritage data. We thank the reviewer once again for these valuable observations, which have helped us clarify both the scope and the intended contribution of the manuscript.

# Reviewer report: Reviewer response for version 2

**DOI:** 10.21956/openreseurope.25768.r73893

**Published:** 2026-05-25

**Authors:** Rich Potter

**Affiliations:** 1 University of Gothenburg, Gothenburg, Sweden

Much more readable than the first draft and ready for indexing with some very minor suggestions.

1. The first sentence in the abstract is not quite right. Does the Côa Valley Archaeological Park hold the data or is it the 2ArchIS database?  This needs to be reworded.
2. in the background you only talk about Palaeolothic art, but you have worked with only the Iron Age material.
3. It would be good to include a definition of triples in your glossary
4. It might be an idea to be explicit about how many of the 1200 sites you use in your project.

Glossary has otherwise worked well, and new images are an improvement.

Is the study design appropriate and does the work have academic merit?

Yes

Is the work clearly and accurately presented and does it cite the current literature?

Partly

If applicable, is the statistical analysis and its interpretation appropriate?

Not applicable

Are all the source data underlying the results available to ensure full reproducibility?

Yes

Are the conclusions drawn adequately supported by the results?

Yes

Are sufficient details of methods and analysis provided to allow replication by others?

Partly

Reviewer Expertise:

Rock Art, Digital Archaeology, 3D Documentation

I confirm that I have read this submission and believe that I have an appropriate level of expertise to confirm that it is of an acceptable scientific standard.

# Response

**Published:** 2026-06-29

**Authors:** Ana BAPTISTA

**Affiliations:** Information Systems, Universidade do Minho Centro ALGORITMI, Guimaraes, Braga, Portugal

We are pleased to submit the revised version of our manuscript. We are very grateful to the reviewer for the time and effort he invested in evaluating our manuscript. We highly appreciate his thorough review and positive guidance, which have greatly helped us enhance both the scientific quality and the readability of this work. Below, we address the reviewer's comments and describe the changes made.

1. "The first sentence in the abstract is not quite right. Does the Côa Valley Archaeological Park hold the data or is it the 2ArchIS database?  This needs to be reworded."
2. "In the background you only talk about Palaeolothic art, but you have worked with only the Iron Age material."

**Response:** We agree that the original wording could create ambiguity regarding the repository of the archaeological data. To clarify this point, the abstract has been revised to explicitly identify the University of Minho’s 2ArchIS database as the platform where the archaeological documentation of the Iron Age engravings is stored and managed. This revision distinguishes the role of the Côa Valley Archaeological Park as the institution responsible for preserving the engraved rocks from that of the 2ArchIS database as the repository of the associated archaeological records.

**revised text** The Côa Valley Archaeological Park, a UNESCO World Heritage, preserves more than 1,200 engraved rocks spanning a long chronological sequence from Prehistory to the Contemporary period. Among these, Iron Age engravings form a significant secondary corpus with no known parallel in the Iberian Peninsula. The archaeological documentation of these engravings is stored and managed in the University of Minho’s 2ArchIS database. This article presents the process of converting these previously restricted archaeological records into an openly accessible dataset, with the aim of improving data sharing, reproducibility, and future research on Iron Age rock art.

1. "It would be good to include a definition of triples in your glossary"

**Response:** We included in the glossary the following entry: “Triple - In this context, 'triple' refers to an RDF triple (or RDF statement), which consists of three elements: subject, predicate, and object—or, in alternative terminology: resource, property, and value. A triple “expresses a relationship between two resources. The **subject** and the **object** represent the two resources being related; the **predicate** represents the nature of their relationship” ^72^ . Whenever possible, persistent IRIs [26] are used to represent and uniquely identify the subject, predicate, and object. For example, the subject can be represented by [https://doi.org/10.34622/datarepositorium/NY5JQS], the predicate by [https://ariadne-infrastructure.eu/aocat/1.1/has_description], and the object can be the literal string "Cavaleiro em combate", which translates to 'Knight in combat'. This means that the description of the resource identified by https://doi.org/10.34622/datarepositorium/NY5JQS is ‘Cavaleiro em combate’."

1. "It might be an idea to be explicit about how many of the 1200 sites you use in your project."

**Response:** We agree that the scope of the dataset should be stated more explicitly. To address this point, the Introduction has been revised to clarify that, although the Côa Valley Archaeological Park contains more than 1,200 documented engraved rocks, the present dataset focuses on a specific subset of Iron Age rock art. The revised text now specifies that the project concentrated on three areas: Vermelhosa (10 engraved rocks), Vale de José Esteves (4 engraved rocks), and Meijapão (3 engraved rocks), corresponding to a total of 17 engraved rocks. This addition makes the scale and limits of the dataset clearer for readers. **revised text** Introduction The Côa Valley (Côa Valley Archaeological Park), located in the Guarda district of Portugal, covers a vast area with more than 1,200 engraved rocks. It is internationally recognised as the largest open-air ensemble of Palaeolithic rock art in the world and has been inscribed on the UNESCO World Heritage List since 1998. While Palaeolithic engravings constitute the dominant component of the rock art record, the valley also preserves later phases of occupation, including Iron Age engravings, which represent the second most frequent chronological phase. ^12, 13 ^ Within this broader archaeological context, the Open Access Rock Art Repository project (RARAA - Repositório de Arte Rupestre de Acesso Aberto), funded by the Portuguese Foundation for Science and Technology (FCT), focused specifically on Iron Age rock art, aiming to improve knowledge of both the engravings and the communities that produced them. Of the approximately 1,200 engraved rocks currently documented in the park, Iron Age engravings form a relatively small but archaeologically significant subset of the record. Within this subset, the project concentrated on three areas: Vermelhosa (10 engraved rocks), Vale de José Esteves (4 engraved rocks), and Meijapão (3 engraved rocks). Consequently, the dataset worked in RARAA corresponds to 17 engraved rocks from three Iron Age rock art areas, representing a small but well-defined subset of the broader rock art record of the Côa Valley. All data gathered by the RARAA project is stored in a relational database called 2ArchIS. While this database serves the project and the Archaeology Unit of the University of Minho well, the team sought to connect their data with those of other research groups and, conversely, to make it available for use and reuse by researchers and the wider public. In short, the goal was to make the data open and semantically interoperable—that is, to publish it as Linked Open Data (LOD). This led to the pilot initiative presented here, in which the Vermelhosa dataset was converted and published as LOD, establishing a workflow that can be extended to the data of the remaining areas. Once again, thank you for your review, which helped us improve the article.

# Reviewer report: Reviewer response for version 1

**DOI:** 10.21956/openreseurope.23694.r70889

**Published:** 2026-03-26

**Authors:** Rich Potter

**Affiliations:** 1 University of Gothenburg, Gothenburg, Sweden

This is an interesting, if at times impenetrable, article relating to the creation of a database for the curation and FAIRification of rock art motifs in Portugal. The article is heavily technical in places, and covers the reasoning behind decisions made during its creation in detail. I am writing this review from the perspective of an end user as someone who works extensively with rock art. It is my opinion that the article should be indexed, but it needs revision first.

 The overall content of the article is good, but I would suggest that the introduction and related work section need to be revised. A lot of the concepts that are introduced seem to not be used later on, or in the case of ShEx, reintroduced fully later on. There are sections where a concept is introduced and then never described in full. For example, linked data on page 3 would benefit from a better description than the quote provided. It would be good if these descriptions used simplified terminology or were expanded upon for a wider audience. You also need to introduce and explain terms like triplestore.

 The main issue for me was that there are too many acronyms throughout the article, which makes it confusing in places, particularly the introduction and related work. This can be alleviated by perhaps removing some terms, e.g. CH should just be cultural heritage. A glossary, or table of acronyms would help improve readability greatly. Several of the acronyms are never introduced, e.g. TGN, CIDOC-CRM, and even the project name RARAA. CIDOC-CRM is frequently written as CIDOC CRM. Towards the end of the article, some of the acronyms are dropped, e.g. LOD becomes Linked Open Data from the conclusion onwards.

 I missed a description of what the intent of the database is. Is it just for data collection/dissemination or are there other reasons behind its creation? Has it already been used for something?

 It is admirable that you have made everything open, however, I was unable to find any description of how to reuse any of the material. It might be an idea to include a readme somewhere. How do you anticipate it will be used?

 While it is obviously not the main theme of the article, it would be good if you expanded a little on the fieldwork on page 3 as the sentence is a little bit standalone. Since this is the basis of where the data in the database comes from, it would be good to know what sort of fieldwork was done.

 Images are generally good, though figure 5 could perhaps benefit from being rotated as it is unreadable on paper. Figure 2 could be improved by moving the labels, if possible, though this is a minor issue.

Is the study design appropriate and does the work have academic merit?

Yes

Is the work clearly and accurately presented and does it cite the current literature?

Partly

If applicable, is the statistical analysis and its interpretation appropriate?

Not applicable

Are all the source data underlying the results available to ensure full reproducibility?

Yes

Are the conclusions drawn adequately supported by the results?

Yes

Are sufficient details of methods and analysis provided to allow replication by others?

Partly

Reviewer Expertise:

Rock Art, Digital Archaeology, 3D Documentation

I confirm that I have read this submission and believe that I have an appropriate level of expertise to confirm that it is of an acceptable scientific standard, however I have significant reservations, as outlined above.

# Response

**Published:** 2026-04-24

**Authors:** Ana BAPTISTA

**Affiliations:** Information Systems, Universidade do Minho Centro ALGORITMI, Guimaraes, Braga, Portugal

We appreciate your valuable input and the thorough review of our work. For clarity, we have addressed your requests by organizing them into the following six points.

 1 – Define better “Linked Data”, triplestore and other concepts for a vaster audience. We improved the definitions of the following terms: Linked Data, Triplestore, Application Profile, RDF, RDFS, SKOS, Values Vocabulary, Properties and Classes vocabulary (sometimes called ontology). All these terms were put into the glossary before the Introduction and their definitions were removed from the body of the text.

 2 – Use less acronyms. While there are some acronyms that can be not used (e.g., CH for cultural heritage), others are better recognized as acronyms than by the expanded version (e.g., SKOS, RDF, FAIR, or CARE). We, therefore, alleviated the following acronyms: CH – Cultural Heritage RDB – Relational Database UAUM – Archaeology Unit of the University of Minho LOV – Linked Open Vocabularies EDM – Europeana Data Model DWBP – Data on the Web Best Practices And other acronyms that were expanded once and never again used.

 3 - Expand – TGN, CIDOC-CRM (or CIDOC CRM?), RARAA and verify all other acronyms. CIDOC CRM does not have a hyphen in the official documentation, so we have amended the sole occurrence in which a hyphen was used. The acronym was already expanded in the text. TGN was also indeed expanded, but the acronym was not related to the expanded version, which we corrected. We also expanded the RARAA acronym and verified all the acronyms.

 4 - It might be an idea to include a readme somewhere. How do you anticipate it [the data] will be used? Under the Conclusions and Future Work, we included a subsection with the following text: “Reuse and access guide All outputs produced in this work are openly available for access and reuse via dataRepositóriUM (DOI-identified deposits) and via the project triplestore (SPARQL endpoint). To make reuse easier—especially for users unfamiliar with RDF or SPARQL, we provide a README document (included in the main deposit record) that explains what each artifact contains and how they relate (DCTAP application profile , ShEx validation shapes, RDF instance data; plus the RDFS namespace schema and the SKOS controlled vocabulary). The materials can be reused in at least three ways. First, as a research dataset, users may download the RDF files (RDF/XML or Turtle) and query them locally, or query the online triplestore via SPARQL, for example to list motifs and their descriptions, retrieve motifs by typology/chronology/technique, explore represented parts, or analyze motif participation in scenes. Second, as reusable semantic specifications, other teams may adopt the DCTAP and the SKOS vocabulary to describe compatible rock art resources and validate their RDF against the ShEx schema before publication or integration. Third, as interoperability building blocks, data managers and developers may reuse the RARAA namespace schema (RDFS) and align local terms and identifiers to facilitate interlinking and enrichment with other datasets. The README provides a “start here” checklist, indicates recommended download formats, and includes a small set of example SPARQL queries and validation instructions to support immediate adoption.”

 5 – Improve Figure 5. Figure 5, originally an automated tool output, has been replaced with a schematic representation. This version incorporates illustrative renderings of specific motifs to enhance interpretative clarity and provide better visual context for the underlying RDF structure.

 6 – Improve Figure 2 (move the labels). Figure 2, originally an automated tool output, has been replaced with a schematic representation.

# Reviewer report: Reviewer response for version 1

**DOI:** 10.21956/openreseurope.23694.r70881

**Published:** 2026-03-20

**Authors:** Philip Riris

**Affiliations:** 1 Bournemouth University, Poole, UK

The authors report on recent work that attempts to transition a database of rock art sites from the Vale do Coa (Portugal), resolved to the motif level, into a Linked Open Data format. As I understand it, the manuscript predominantly focuses on the process and methodological justifications underpinning the end-product, i.e. a complete LOD dataset. My comments chiefly concern: 1) the accessibility of the text itself, and 2) the underlying RARAA dataset. Not being a domain expert in LOD or data interoperability, I am writing from the point of view of a prospective end-user and invite the authors to consider the accessibility of the text to the non-expert. I appreciate that the manuscript concerns a specialist subject, however, as the journal has a general and broad readership, I am emphasising this element more than I perhaps would in a specialist disciplinary journal such as *Journal of Computer Applications in Archaeology* or *Advances in Archaeological Practice*. 

 In the first instance, the introduction would especially benefit from re-writing in layman's terms. There are 14 acronyms introduced on a single page. Although some are familiar or obvious, e.g. *UNESCO* or *FAIR, *the meaning and overall flow of the text is disrupted by this much information. Additionally, there is liberal use of direct quotes throughout the text (usually definitions, e.g. 'open data', fifth paragraph). **T** he authors may consider making a new table or text box for the manuscript which provides a glossary of terms, and place it in the introduction **.** This would enable the authors to foreground the meaning and significance of their text, rather than losing the reader in a sea of acronyms. The existing text would necessarily be shortened, allowing space for more plain-language explanation of *why* this work is being performed. Even as a proponent and practitioner of Open Science, who understands the fundamental impetus for this sort of work (and I am deeply appreciative of its development in a rock art context), the justification gets lost. In summary: the introduction especially needs strengthening by simplifying it. The authors should consider this general advice for the text as a whole **, **but it's particularly noticeable in the introduction. 

 Regarding the second point, I find there is a disparity between the reported 1200 engraved outcrops/boulders in the Coa Valley Park, and what the 2ArchIS database on http://www.raraa.pt/ actually contains. I apologise to the authors if I have misunderstood, or if I am attempting to access the wrong dataset. Nevertheless, the fact that I cannot tell where to look or what to look for is, in itself, a justification for a clearer explanation and signposting of the underpinning archaeological evidence (i.e. the actual transcendental value of this research). It only really becomes clear in the final sections that primary rock art imagery (sketches, photos, tracings, rubbings) is *not* included as part of the LOD (to my knowledge). Rather, it is a series of data tables recording the various interpretations *of* the underlying imagery. One of the unspoken principles of Open Data is "trust, but verify" and without backlinks to the original imagery this is not possible. The imagery apparently *is* available ( here) but the Data Availability statement does not contain reference to it. The LOD product is impressive, useful, and worth publishing but end-users will always ultimately want to return to the original data (for example for re-interpretation or comparative work), which currently does not seem to be possible.

 Aside from this, the "atlas" linked above only contains sketches (not original images) of Iron Age rock art and I therefore find it misleading to make an implicit link between the entire corpus of Coa Valley rock art (which is extensive, varied, and multi-period) and the rather constrained sample of sites within the U of Minho repository. It must be made clearer in the introduction that the focus is *specifically on *Iron Age rock art here - it is currently mentioned once in passing in the introduction and in the conclusion. How it compares in terms of number of sites, outcrops, motifs, themes, etc. with the more well-known Palaeolithic engravings should be considered, so that readers have a better handle on the content of the LOD. 

**Other comments**

 On page 6 ( **section Linked data development...**), you must identify which projects you are talking about. "Some of these project" [...] "others" [...] "Many projects" is not specific enough, because multiple examples are provided in the subsequent text, and it is not clear to me which examples relate to, e.g. extensive use of ontologies versus, e.g. value vocabularies. Rewrite this for more clarity. 

 Page 7, (section **The 2ArchIS relational database**) needs, at a minimum, citations of supporting literature on the aspects of society and environment that rock art can inform us about.

 Page 17, ( **The triplestore**), it should be "6085" not "6.085".

 Page 19, second to last paragraph: "humans" seems to be a false friend from "homens" - replace with "users", "researchers", or "people".

Is the study design appropriate and does the work have academic merit?

Yes

Is the work clearly and accurately presented and does it cite the current literature?

Yes

If applicable, is the statistical analysis and its interpretation appropriate?

Not applicable

Are all the source data underlying the results available to ensure full reproducibility?

No

Are the conclusions drawn adequately supported by the results?

Yes

Are sufficient details of methods and analysis provided to allow replication by others?

Partly

Reviewer Expertise:

Rock art, computational archaeology, simulation & modelling, historical ecology.

I confirm that I have read this submission and believe that I have an appropriate level of expertise to confirm that it is of an acceptable scientific standard, however I have significant reservations, as outlined above.

# Response

**Published:** 2026-04-24

**Authors:** Ana BAPTISTA

**Affiliations:** Information Systems, Universidade do Minho Centro ALGORITMI, Guimaraes, Braga, Portugal

We appreciate your valuable input and the thorough review of our work. For clarity, we have addressed your requests by organizing them into the following nine points. 1 – Glossary A glossary was added before the Introduction with the following terms:

- Classes and properties vocabulary
- Linked Data
- Linked Open Data
- Metadata Application Profile
- Resource Description Framework (RDF)
- RDF Graph
- RDF Schema (RDFS)
- Shape Expressions (ShEx)
- Simple Knowledge Organization System (SKOS)
- SPARQL
- Triplestore
- Values vocabulary

2 – Alleviate the acronyms. While there are some acronyms that can be not used (e.g., CH for cultural heritage), others are better recognized as acronyms than by the expanded version (e.g., SKOS, RDF, FAIR, or CARE). We, therefore, alleviated the following acronyms: CH – Cultural Heritage RDB – Relational Database UAUM – Archaeology Unit of the University of Minho LOV – Linked Open Vocabularies EDM – Europeana Data Model DWBP – Data on the Web Best Practices And other acronyms that were expanded once and never again used. 3 – Explain better why this work is being performed. With the glossary and the need to explain better some parts of the work, we reformulated the whole Introduction. The following paragraphs are part of this reformulation: “All the data gathered by the RARAA project is stored in a relational database called 2ArchIS. Although the relational database is beneficial and widely used by the RARAA project and the Archaeology Unit of the University of Minho team, the researchers would like to be able to relate their data to those of other research teams and, conversely, to be able to share their data with other researchers or interested citizens for use and reuse. In other words, the RARAA team wanted its data to be open and semantically interoperable, that is, to be Linked Open Data (LOD). This need aligns with broader developments in the digital transformation of cultural heritage. In recent years, global initiatives promoting open, structured, and reusable data have significantly influenced the digital transformation of cultural heritage. The digital transformation of cultural collections has strengthened preservation practices and opened new opportunities for research, education, and public engagement. ^3^^, ^^4^ This paradigm shift has been accompanied by international initiatives that promote open, structured data, ensuring that heritage knowledge remains accessible and reusable sustainably. Movements such as Open GLAM and Collections as Data advocate for the responsible sharing and ethical reuse of cultural data. This approach is exemplified by the principles articulated in documents such as the Vancouver Statement on Collections as Data. ^5^ These initiatives align closely with the FAIR principles (Findable, Accessible, Interoperable, and Reusable), ^6^ which enhance machine-actionable data discovery and usage while supporting human reuse, and with the CARE principles (Collective Benefit, Authority to Control, Responsibility, Ethics), which provide a foundational approach to Indigenous Data Governance. ^7^ In this context, the main goal of this work was therefore to transform data in a relational database into LOD and make it available for use and reuse, adhering to principles and practices that promote structured, reusable, and interconnected data, including the FAIR principles and the World Wide Web Consortium (W3C) Data on the Web Best Practices ^1^ ^5^ ^,1^ ^6^. The expected outcome is to enable integration of RARAA’s data with datasets from other research teams and to support broader reuse by the academic community and the general public. By enabling such integration and comparison, we aim to generate new insights into Iron Age rock art that would not be possible when data remains confined to isolated systems, often called data silos. To achieve this, we designed an application profile and related artifacts and then used them to expose 2ArchIS data as LOD.“ In another part of the text, we clarified again why it is important, in our view, to have the data open as Linked Data: “In addition, it presents in detail the process that led to making this data openly available from an existing relational system, with the goal of supporting reuse, extension, and interlinking by the wider community.” 4 – Disparity between the 1200 engraved rocks and the number of motifs in the 2ArchIS database and  the triplestore. Another part of the reformulation: “Within this broader archaeological context, the Open Access Rock Art Repository project (RARAA - Repositório de Arte Rupestre de Acesso Aberto), funded by the Portuguese Foundation for Science and Technology (FCT), explored the Côa Valley in search of Iron Age rock art, aiming to increase knowledge of the art and the communities that created it. Of the approximately 1200 engraved rocks currently known in the park, a significant proportion date from the Iron Age, the second most represented chronological period in the Côa Valley. Within this subset of Iron Age rock art, the project focused on documenting the engravings from three specific areas: Vermelhosa, Vale de José Esteves, and Meijapão . Consequently, the data presented in this work correspond to a small subset of the extensive rock art record of the park.”  5 – Section Linked Data Development – Clarify: “Some of the projects” (…) “others” (…) “Many projects”. – We replaced that introductory paragraph with the following: “The transition from legacy cultural heritage data to the Semantic Web has been addressed through various methodological lenses. While some approaches prioritize the preservation of metadata richness through multi-step ingestion processes, others focus on the scalability of database migrations or the agility of ontology design. The following projects illustrate the diversity of frameworks developed to bridge the gap between heterogeneous data sources and ontological structures.“ 6 - Section “The 2ArchIS Relational Database” – needs, at a minimum, citations of supporting literature on the aspects of society and environment that rock art can inform us about. We added the following text: “In this context, 2ArchIS ^69^ was created by the Archaeology Unit of the University of Minho as an operational information system to support archaeological work from field recording to post-fieldwork analysis and reporting. Its primary intent is to ensure consistent data capture in the field and in the office, to centralize and normalize records produced by different team members, and to maintain long-term institutional memory of projects. Beyond data entry, the system supports day-to-day research workflows by enabling browsing and filtering of records, production of inventories and summary tables for reports, and systematic association of archaeological entities with documentation (bibliography, photographs, drawings, cartography, and 3D models). In the specific case of rock art, such structured recording is particularly relevant given that these datasets constitute a primary source for interpreting past social practices, symbolic systems, and the construction of cultural landscapes, as well as human–environment interactions reflected in the representation and spatial distribution of motifs ^71,72^. For the RARAA project, 2ArchIS [8] has served as the authoritative source for recording and relating archaeological sites, rocky outcrops, motifs, represented parts, scenes, and associated documentation, supporting both ongoing research and project outputs. For dissemination and preservation purposes, exported records have also been deposited in an open repository with persistent identifiers, while 2ArchIS remained the primary environment for data creation and curation.“ With references (69,71,72): (69) Botica N, Magalhães F, Machado D, Fontes L. Del sistema de información 2ArchIS al DataRepositóriUM: el estudio de caso de hallazgos arqueológicos. Cuad Arqueol Univ Navar. 2021 Apr 14;29:381–96. doi:10.15581/012.29.005 (71) Bettencourt AMS, Santos-Estevez M, Sampaio HA, Cardoso D. Prehistoric Rock Art and Landscape: the outcrops and its forms were certainly not simple carving surfaces. In: Ribeiro C, Martins RN, editors. I International Seminar on Art and Landscape [Internet]. Guimarães, Portugal: Laboratório da Paisagem; 2017. p. 137–53. Available from: http://www.labpaisagem.pt (72) Cruz Berrocal M, Vicent García J. Rock art as an archaeological and social indicator: The neolithisation of the Iberian Peninsula. J Anthropol Archaeol. 2007 Dec;26(4):676–97. doi:10.1016/j.jaa.2007.02.003 7 – The triplestore – 6.085 -> 6085 – Done. 8 - Page 19, second to last paragraph: "humans" seems to be a false friend from "homens" - replace with "users", "researchers", or "people". We used the term “interface for humans” as opposition of the SPARQL endpoint, which is an interface for machines (and specialized humans). The term “human” in this context is often used, for instance, in Human-Computer Interaction (HCI). 9 – “Are all the source data underlying the results available to ensure full reproducibility? No“ We made available the source data in the data repository as an XML file, exported from the **2ArchIS** database at the time of the study. We have also provided the formal citation and the corresponding reference for this file. The reference is: Botica N. 2ArchIS XML Data Export (RARAA Project) [Internet]. Repositório de Dados da Universidade do Minho; 2026 [cited 2026 Apr 15]. Available from: https://doi.org/10.34622/datarepositorium/KZEINV doi:10.34622/DATAREPOSITORIUM/KZEINV
